# Food or just a free ride? A meta-analysis reveals the global diversity of the Plastisphere

**DOI:** 10.1038/s41396-020-00814-9

**Published:** 2020-11-02

**Authors:** Robyn J. Wright, Morgan G. I. Langille, Tony R. Walker

**Affiliations:** 1 School for Resource and Environmental Studies, Dalhousie University, Halifax, Canada; 2 Department of Pharmacology, Faculty of Medicine, Dalhousie University, Halifax, Canada; 3 Department of Microbiology and Immunology, Dalhousie University, Halifax, Canada

**Keywords:** Microbial ecology, Soil microbiology, Water microbiology, Microbial ecology, Microbiome

## Abstract

It is now indisputable that plastics are ubiquitous and problematic in ecosystems globally. Many suggestions have been made about the role that biofilms colonizing plastics in the environment—termed the “Plastisphere”—may play in the transportation and ecological impact of these plastics. By collecting and re-analyzing all raw 16S rRNA gene sequencing and metadata from 2,229 samples within 35 studies, we have performed the first meta-analysis of the Plastisphere in marine, freshwater, other aquatic (e.g., brackish or aquaculture) and terrestrial environments. We show that random forest models can be trained to differentiate between groupings of environmental factors as well as aspects of study design, but—crucially—also between plastics when compared with control biofilms and between different plastic types and community successional stages. Our meta-analysis confirms that potentially biodegrading Plastisphere members, the hydrocarbonoclastic *Oceanospirillales* and *Alteromonadales* are consistently more abundant in plastic than control biofilm samples across multiple studies and environments. This indicates the predilection of these organisms for plastics and confirms the urgent need for their ability to biodegrade plastics to be comprehensively tested. We also identified key knowledge gaps that should be addressed by future studies.

## Introduction

An estimated total of 7 billion metric tons of plastic waste has now been produced globally while approximately only 9% of this is recycled and 79% has been discarded in landfills or the environment [1]. In marine and other aquatic environments, plastics cause a range of negative environmental impacts: directly, through entanglement [2, 3] or ingestion [4–8], or indirectly, through the transfer of toxic chemicals [9, 10]. Despite that the majority of marine plastic waste originates from land, less is known about the impacts of plastic waste in terrestrial ecosystems [11], and there are numerous factors that can affect the fate, transport, and impacts of plastics in all biospheres.

These factors include transport by air [12], rain [13], rivers [14] and currents [15], (de)sorption of chemicals [16], photo- or mechanical degradation or fragmentation [17] and microbial colonization and possibly degradation [18]. Deleterious impacts of plastics [6], their potential to transport invasive species across entire ocean basins [3, 19–21] and their biodegradation by isolated bacteria and fungi have been studied for over half a century [22]. The microbial communities colonizing plastics—commonly termed the “Plastisphere” [23]—however, have only been specifically investigated more recently. A call for research into the interactions between microorganisms and plastics at the beginning of 2011 marks almost a decade of Plastisphere research [24]. On 5 January, 2020 there were 50 publications that studied the Plastisphere using Illumina Next Generation Sequencing (NGS) methods. Although this is an emerging area of research, most Plastisphere studies have been focussed on the marine environment.

Microbial members of the Plastisphere have been found to be: (i) different from communities that colonize other surfaces [25–27]; (ii) not different from communities that colonize other surfaces [28]; (iii) only different from communities colonizing other surfaces under specific environmental conditions [29, 30] or at specific time points [31]; (iv) more diverse than other microbial communities [32]; (v) less diverse than other microbial communities [33, 34]; (vi) potentially degrading the plastics that they colonize [35, 36]; (vii) capable of degrading plastic additives [33, 37, 38]; and (viii) pathogenic and/or carrying antimicrobial resistance genes [39–42]. The marine Plastisphere has been heavily reviewed within the last year, e.g., [18, 43–46], and there are also several recent reviews on plastic biodegradation, e.g., [47–49]. However, definitive answers on the metabolic capabilities of the Plastisphere or the factors that drive its formation and composition are unknown. Indeed, a re-analysis of a small subset of Plastisphere studies (*n* = 5) by Oberbeckmann and Labrenz [45] revealed that salinity along with other environmental factors appeared to have a larger effect on community composition than substrate.

To date, no large-scale meta-analysis of the Plastisphere has been conducted, despite that all NGS data collected are theoretically comparable. This is likely because these data are not always deposited in publicly accessible databases (as best practice would dictate). In addition, the use of different methods for processing these data means that direct comparisons have not been possible without substantial bioinformatic analyses. In this study, we conducted a re-analysis of all studies from 2010 to 2019 (for which sequencing data were already accessible or were made available upon request) that use Illumina NGS to characterize the Plastisphere using the 16S rRNA gene. We aimed to determine whether taxa identified as potential plastic biodegraders or potential pathogens -and that were higher in abundance on plastics than other samples- were significant across multiple studies and environments. We then investigated which environmental and methodological factors were shaping the Plastisphere. We classified all sequences from these studies to amplicon sequence variants (ASVs) and used a phylogeny-based method [50] to overcome the problems presented by the use of different primer pairs. This allowed us to identify the common taxa between these studies and to use random forest models to draw conclusions on the over-arching factors that shape the Plastisphere.

## Materials and methods

### Experimental design

A literature search was performed on 5 January, 2020 using the search terms “Plastics plastisphere”, “Plastics microbial community”, and “Plastics microbial degradation” in both the Web of Science Core Collection and Science Direct (Supplementary Table 1A). The search was limited to studies that fit the following criteria: (i) were published between 2010 and 2019; (ii) had original data (iii) characterize the biofilm formed on nonbiodegradable plastics; and (iv) use Illumina NGS. This resulted in 50 studies for inclusion in this meta-analysis (Supplementary Table S1B); 41 studies that characterized only the 16S rRNA gene [25, 27–33, 35, 38, 41, 42, 51–79], two studies that characterized only the 18S rRNA gene [34, 80], two studies that characterized the 16S and 18S rRNA genes [26, 81], two studies that characterized the 16S and ITS2 rRNA genes [82, 83], two studies that used shotgun metagenomics [84, 85], and one study that used shotgun metagenomics and characterized the 16S rRNA gene [86].

Of these studies, 34 had sequencing data that were already publicly accessible. Requests for raw sequencing data and metadata were made to the corresponding and first authors of the remaining 16 studies, resulting in the provision of these data for a further five studies. This resulted in 39 studies with datasets that were available for inclusion: 31 that characterized only the 16S rRNA gene [25, 27, 28, 30–33, 35, 38, 52, 53, 55–59, 61, 62, 64–66, 68, 69, 71–76, 78, 79], two that characterized only the 18S rRNA gene [34, 80], two that characterized the 16S and ITS2 rRNA genes [82, 83], two that used shotgun metagenomics [84, 85], one that characterized the 16S and 18S rRNA genes [26], and one that used shotgun metagenomics and characterized the 16S rRNA gene [86] (Supplementary Table S1B). Relatively few studies focussed on the 18S and ITS2 rRNA genes and these used primers that targeted different regions and were from different environments. We therefore focussed on the 16S rRNA gene here, meaning that we included a total of 35 studies [25, 27, 28, 30–33, 35, 38, 52, 53, 55–59, 61, 62, 64–66, 68, 69, 71–76, 78, 79, 82, 83, 86] with 2,229 samples between then. Publicly accessible data containing sequencing reads for the remaining studies were downloaded (primarily from the NCBI SRA database) and, if necessary, files were converted to match a format that was compatible with QIIME2 (i.e., GNU zipped FASTQ files). Additional requests for raw sequencing data were made to the authors of studies where the forward and reverse reads were already joined, or the primers were already removed (full details can be found in Supplementary Table S1B). All available metadata were collected and supplemented with any additional information present in the supplementary information of published studies (Supplementary Table S2). Where details of salinity and temperature were not given, these were estimated based on typical characteristics in these areas at the time of year the samples were collected. If no light regime was specified, ambient light was assumed. If sample names were not given that could be matched between supporting information of the paper and metadata given to the NCBI SRA or plastic type was not determined, these were classified as “unknown plastic”.

We added metadata categories that were not in any of the original studies: (i) environment—each study was classified as terrestrial, marine, freshwater, or aquatic (e.g., the environment was brackish or the experiment was carried out in a different system, such as in mariculture); (ii) whether the study was carried out in a laboratory or in the field; (iii) incubation or collection—whether the plastics used were incubated for a known length of time or were collected after an unknown residence time; (iv) general incubation time—samples were classified as early (≤7 days incubation), late (>7 days incubation), or collection (samples were collected after an unknown residence time); (v) water or sediment—the plastics were from/were incubated either in the water column or on the sea floor/in soil in the terrestrial environment; (vi) source—the material used for microbial community characterization was classified to the general categories of plastic, water, sediment, organic, not plastic (i.e., an inert control surface, such as glass or metal), other or blanks and positive sequencing or methodological controls; and (vii) general plastic type—samples were classified as aliphatic (i.e., PE or PP), other plastic (i.e., plastics that contain other functional groups, e.g., PET, PS, and PVC), unknown plastic (the plastic type was not determined), biofilm (the sample was from a control substrate, such as glass or a leaf), planktonic, or blank (i.e., sequencing or methodological controls).

### 16S amplicon sequence processing (per study)

Data processing followed the standard operating procedure suggested in Comeau et al. [87] and used QIIME2 (2019.10 core distribution [88]). Raw forward and reverse read files were imported to the QIIME2 format after an initial visualization of read quality using the packages FastQC (Babraham Bioinformatics) and MultiQC [89]. Cutadapt [90] was used to remove primers from reads and VSEARCH [91] was used to join paired-end reads. These steps were omitted for samples where primers were already removed or reads were already joined, respectively, and for one study that used three different reverse primers [59]. All low-quality reads were then filtered using default quality thresholds before using Deblur [92] to denoise sequences and resolve ASVs. Deblur was run using trim lengths determined by the read quality for each study. For Frére et al. [59], the ends of reads corresponding to the lengths of primer sequences were also trimmed. The forward reads only were used for several studies where low reverse read quality led to too few reads remaining after running Deblur and for which the forward reads were of high enough quality to be used (full details of the processing steps carried out for each study can be found in Supplementary Table S2).

### Combined processing

All studies were merged using QIIME2’s merge and merge-seqs commands, then classified taxonomically using a classifier trained on the full-length 16S rRNA gene SILVA v132 database [93]. Classified sequences were filtered to remove mitochondria, chloroplasts, those that were unclassified at the kingdom level and those present at a cumulative abundance of ten or fewer. This left a median of 20,284 reads per sample (minimum 2 and maximum 995,391). Samples with <2,000 reads were removed, leaving 2,056 samples and 34 studies, and phylogenetic trees were built using SEPP with a reference phylogeny created using the SILVA v128 database [50].

Custom scripts that wrapped all commands were used to carry out further analyses in R (version 3.6.1) and Python (version 3.8.3) using the data exported from QIIME2 and the packages Biopython [94], csv, itertools, lifelines [95], math, matplotlib [96], numpy, os, pandas [97], pdf2image, pickle, scipy [98], scikit-bio, scikit-learn [99], and sinfo for Python as well as ape [100], compositions [101], dplyr [102], exactRankTests [103], ggnewscale [104], ggplot2 [105], ggtree [106], knitr [107], metacoder [108], microbiome [109], nlme [110], philr [111], phyloseq [112], reticulate [113], and vegan [114] for R. Several normalization methods were used to address the large disparity in the sequencing depths of different samples: (i) samples were rarefied to 2,000 and converted to relative abundance; (ii) samples were converted to relative abundance; (iii) samples were converted to a log scale (with a pseudo count of one); or (iv) samples were converted to a centered log ratio (CLR; with a pseudo count of half of the minimum nonzero count). ASVs with a maximum number of reads below 1% of the median number of reads per sample were removed—below 20 reads for (i) and 202 reads for (ii), (iii), and (iv)—and sequences were agglomerated at a height of 0.1 based on the SEPP insertion tree. This resulted in 4,469 ASVs remaining for (i) and 12,635 ASVs remaining for (ii), (iii), and (iv). For (i), (ii), and (iii), weighted and unweighted uniFrac [115] distances were calculated between all samples while for (iv), the log-ratio transformed data, Aitchison distances (i.e., Euclidean distances of CLR-transformed data) [116] as well as Phylogenetic Isometric Log-Ratio Transformation [111] distances were calculated between all samples. Unless otherwise mentioned, all analyses used the rarefied data.

### Random forest model construction

To determine which taxa were most associated with groupings within each metadata category (e.g., environment, plastic type, salinity, incubation time; *n* = 20 categories), feature selection was performed using random forest models (either classification or regression models for discrete or continuous data, respectively) built using scikit-learn, each with 10,000 estimators and 80 and 20% of samples being used for training and testing, respectively. All taxa were scaled to the maximum value of taxon abundance prior to building the models. These were carried out separately for the taxonomic levels of phylum, class, order, family, genera, species, and ASV. Where groupings within metadata categories for samples were not known, these samples were removed from the model construction. To investigate the effect of normalization method on the selected features, these models were constructed for each of the four normalization methods described above. To determine the taxa that were most associated with different plastic types, random forest classification models (built using the same parameters as above) were constructed separately for each environment (marine, aquatic, freshwater or terrestrial) for samples grouped to general plastic type (as described above). Samples were not further grouped into laboratory and field studies because not all environments included both. These were again constructed for the taxonomic levels of phylum, class, order, family, genus, species, and ASV and for each of the four normalization methods described above. The classification accuracy for each random forest model is defined as the percentage of the time that the model can classify a sample to the correct grouping (e.g., as marine within the environment metadata category) and the feature importance is defined as the proportion that the classification accuracy would decrease without that taxon (feature) present. We calculated concordance (using the Python lifelines package) [95] between the classification accuracies as well as between mean feature importance values to assess the similarity of the results obtained by the random forest models constructed using different normalization methods.

### Tests for differential abundance of taxa

ASVs shared between treatments were calculated for samples grouped by the environment. Differences between samples at early (≤7 days) or late (>7 days) incubation times were determined using Wilcoxon Rank Sum tests within the metacoder R package with holm-bonferroni false discovery rate correction. ANCOM tests for differential abundance (with holm-bonferroni false discovery rate correction) were performed on all taxa (separately for each taxonomic level) for the taxa normalized using rarefaction.

## Results

### Summary of included studies and sequences

**Fig. 1:**
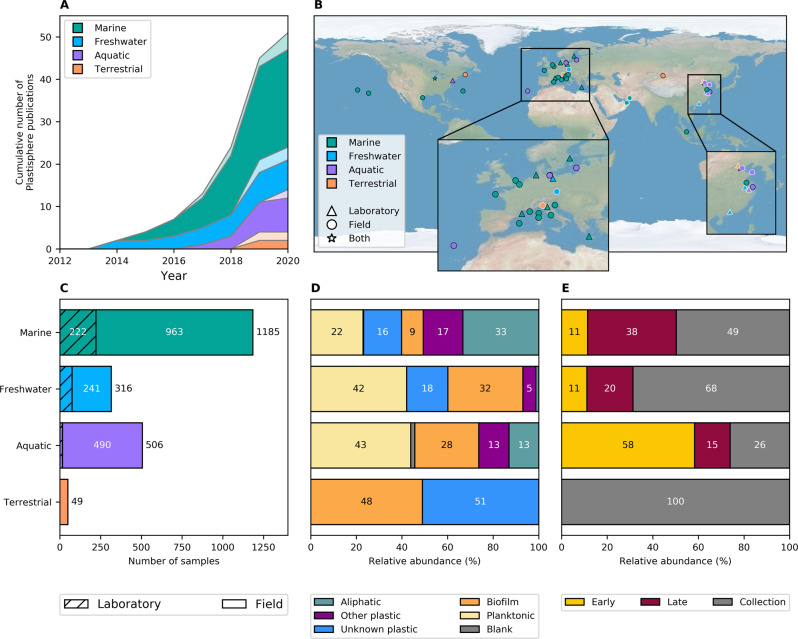
Overview of the studies and samples included in the meta-analysis. Cumulative number of studies per year (**A**), study location (**B**), number of samples (**C**), relative abundance of sample type (**D**), and sample incubation time (**E**) for studies carried out in the marine, freshwater, other aquatic, and terrestrial environments. **A**, **B** Show all 50 studies whereas, **C**, **D**, and **E** show only studies/samples that were included. Studies for which data were not provided (neither publicly available nor provided upon request) are shown with transparent colors in **A** and white marker edges in **B**. Note that those studies shown for 2020 were already in press and available online by 5 January, 2020. See Supplementary Tables S1 and S2 for full details of all studies and samples included.

In this study, we reanalyzed the 16S rRNA gene amplicon sequencing data obtained from 35 studies (Supplementary Section 1). All Plastisphere studies to date have examined data collected in the Northern hemisphere and most of the included studies were conducted in and around Europe (Fig. 1). After removing all samples with below 2,000 reads there were 2,056 samples remaining; 1,185, 316, 506, and 49 samples in the marine, freshwater, aquatic, and terrestrial environments, respectively. One study [64] was removed from further analyses because all samples (*n* = 9) had below 2,000 reads. The abundance of different substratum types depended on the environment and most samples were collected from the field after unknown environmental residence times (Fig. 1).

### Normalization method affects the biological interpretation of results

There was a large disparity in the number of reads and taxonomic richness per sample between different studies (Fig. S1). We therefore investigated several different methods for data normalization (further details are given in the methods section) on random forest classification accuracy and the concordance between taxa identified as important by each of these methods (Supplementary Section 2). These random forest models were constructed for: (i) all 20 metadata categories—including factors such as environment, geographic location, temperature, primer pair, and plastic type to varying degrees of specificity—for each taxonomic level: phylum, class, order, family, genus, species, and ASV (i.e., 560 random forest models were constructed in total, 140 for each normalization method); and (ii) general plastic type within each of the four environments (marine, aquatic, freshwater, and terrestrial) for each taxonomic level: phylum, class, order, family, genus, species, and ASV (i.e., 112 random forest models were constructed in total, 28 for each normalization method). For both sets of random forest models, the models constructed using rarefied data were on average the least accurate (61–67% and 53–67% mean classification accuracy across all metadata categories and all environments for general plastic type, respectively, for the taxonomic levels of phylum-ASV; Figs. S2 and S3) and the models constructed on data transformed to relative abundance were on average the most accurate (82–90% and 72–86% mean classification accuracy across all metadata categories and all environments for general plastic type, respectively, for the taxonomic levels of phylum-ASV; Figs. S2 and S3). Models constructed using the compositionally aware centered log-ratio transformed data were on average 1.1 and 10% (for all metadata categories and all environments for general plastic type, respectively) less accurate than models constructed on data transformed to relative abundance. Feature importance values are the proportion that the classification accuracy decreases without that feature. The features that have importance values of either above 0.01 or 0.005 are the same between all normalization methods across all taxonomic levels. Concordance in feature importance values (whether the ranking of features by their values is the same) was on average 0.94 across all taxonomic levels between the relative abundance, log and CLR-transformed data and 0.78 for the relative abundance, log and CLR-transformed data against the rarefied data (Supplementary Section 2).

Rarefying has previously been found to most effectively account for large differences in library sizes, including lowering the false discovery rate when there are large differences in library sizes [117]. We therefore compromised on lower random forest classification accuracy and used the rarefied data for the remainder of analyses. Most analyses presented here have also been conducted on the data normalized using the other methods, but these results are presented in Supplementary Section 2.

### Diversity within and between studies

**Fig. 2:**
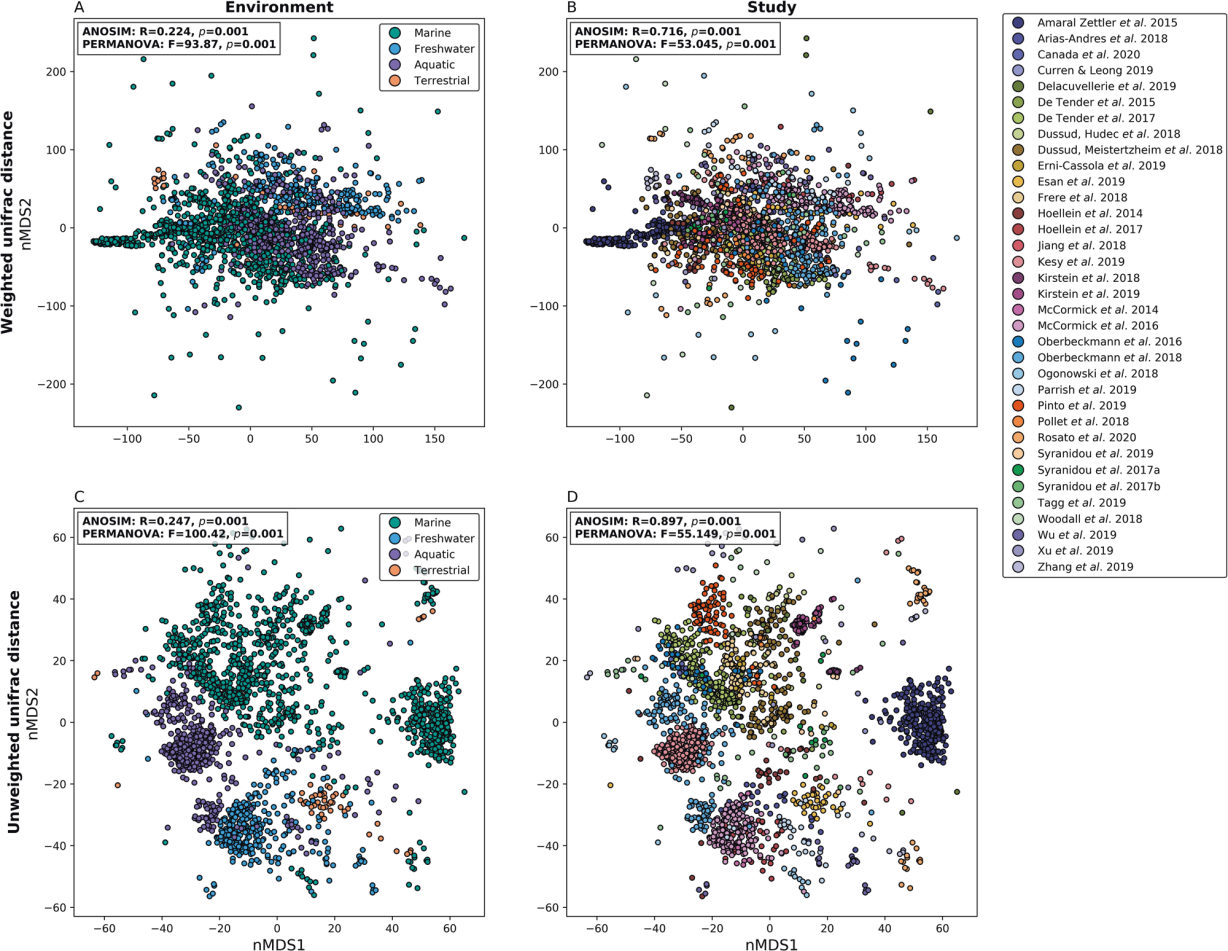
nMDS plots showing uniFrac distance between samples. nMDS plots showing weighted (**A**, **B**) or unweighted (**C,** **D**) uniFrac distance (i.e., accounting for taxon phylogeny with or without taxon abundance, respectively) calculated between all samples and shown in **A** and **C** as samples colored by environment and **B** and **D** as samples colored by study. Results of PERMANOVA and ANOSIM tests for significance between groups are shown on each plot.

**Fig. 3:**
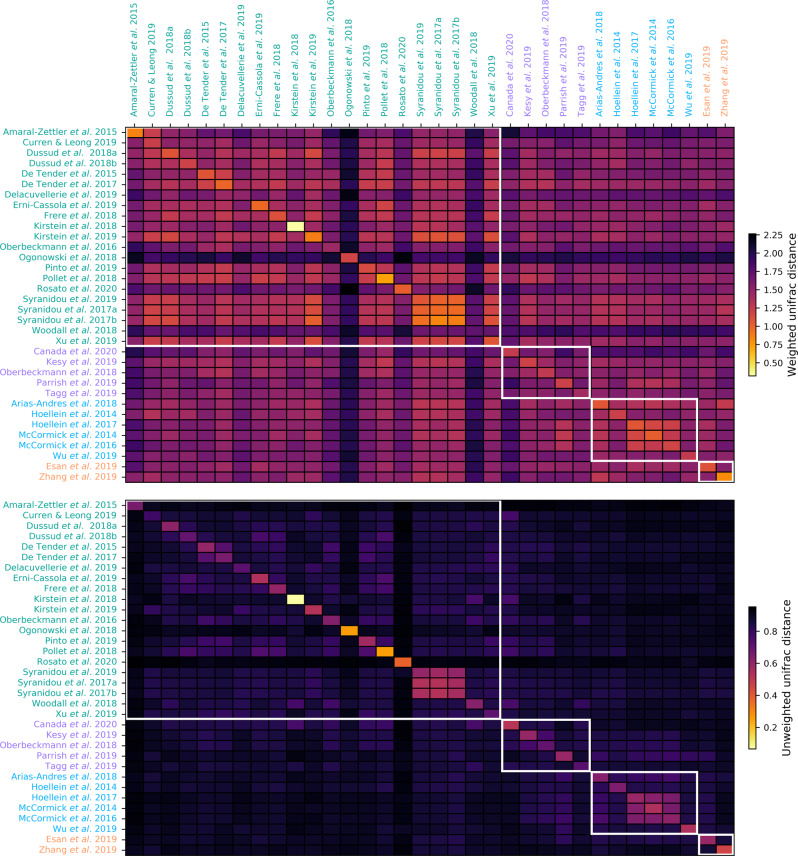
Average similarity between samples within a study *versus* between studies. Average similarity (determined by weighted or unweighted uniFrac; i.e., accounting for taxon phylogeny with or without taxon abundance; top or bottom, respectively) between samples within a study *versus* between studies, with white boxes showing samples grouped by environment. Study names are colored by environment, with green, purple, blue, and orange being for marine, aquatic, freshwater, and terrestrial, respectively (as in Figs. 1 and 2).

Beta diversity analyses (weighted and unweighted uniFrac distance) showed that there were significant differences between samples when they were grouped by study or environment (PERMANOVA and ANOSIM *p* = 0.001; Fig. 2). The largest differences between studies and environments were due to organisms that were present at lower abundances, as evidenced by higher pseudo-F and R statistics with the unweighted (pseudo-F = 100.42 and 55.149 and *R* = 0.247 and 0.897 for environment and study, respectively) than weighted (pseudo-F = 93.87 and 53.045 and *R* = 0.224 and 0.716 for environment and study, respectively) uniFrac distances (Figs. 2 and 3). Studies that were clearly less similar to other studies could be explained by them being laboratory-based [32, 71], focussed on anaerobic rather than aerobic communities [71], collecting samples from the deep sea [76] or sequencing amplicons that did not include the V4 16S rRNA gene region [52, 71]. Those that were particularly similar within [26] or between [72–74] studies could presumably be explained by very long incubation times (above 1 year), leading to community convergence, or having similar experimental setups and inoculums, respectively (Fig. 3).

**Fig. 4:**
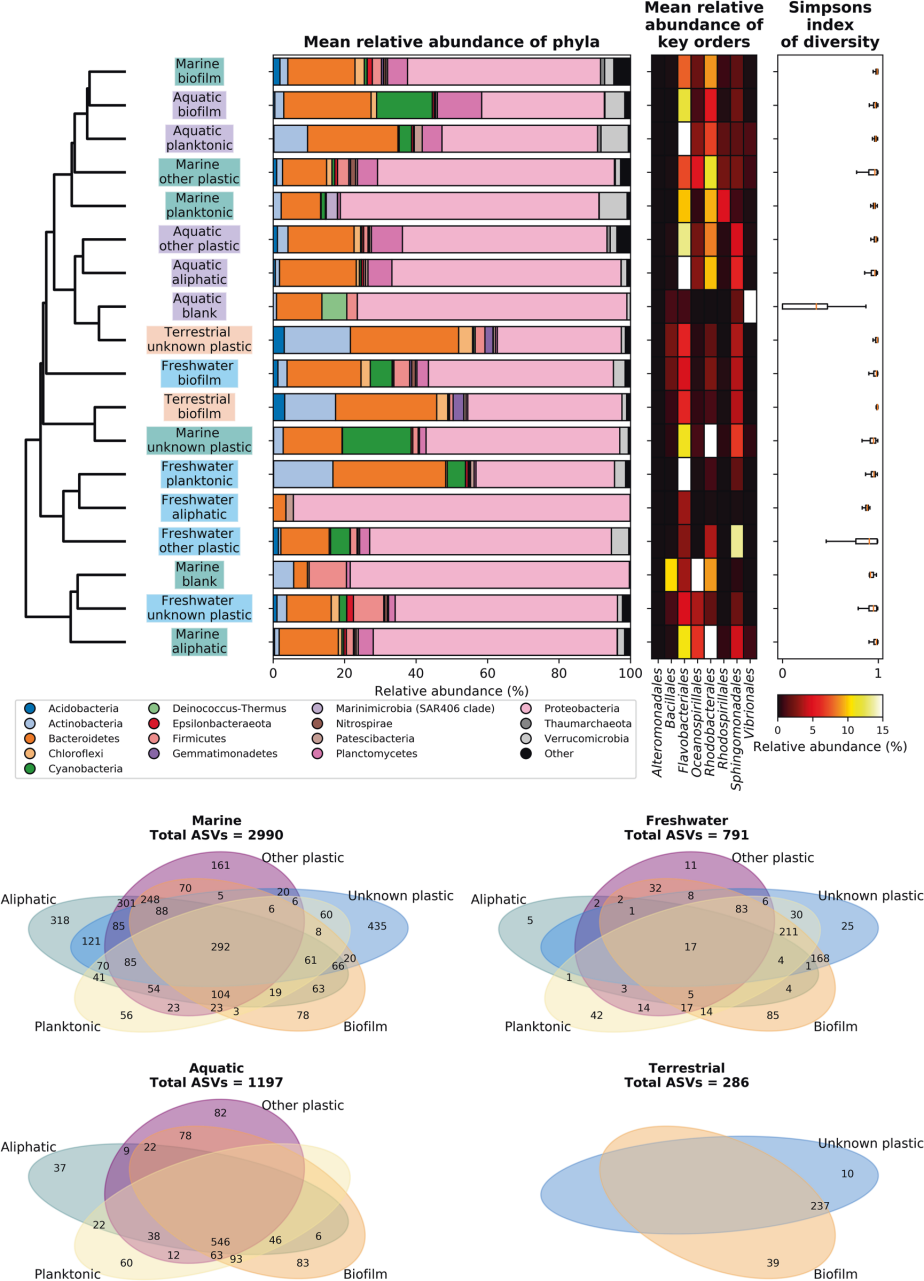
Summary of the composition, diversity and shared ASVs within sample groupings. Similarity of the composition of microbial communities on different substrata in different environments. Samples are grouped by weighted uniFrac distance using ward linkage (dendrogram) between sample types (colored by environment) and mean community composition at the phylum level is shown. Those phyla that are grouped into ‘Other’ are phyla that are <1% mean relative abundance. Mean relative abundance of several orders that have previously been suggested to be associated with plastics and Simpsons Index of Diversity (showing median and interquartile range) for each group is also shown. The number of ASVs that are shared between different substratum types (that are >1% in relative abundance in these samples) is shown at the bottom.

When samples are split to environment and substratum type (i.e., the groupings shown in Fig. 1D), they have a tendency to group by environment, although this is not true for all cases (Fig. 4). The *Proteobacteria* dominate (above 50% relative abundance) in all but the terrestrial environment, freshwater planktonic, and aquatic control biofilm groups, which have larger proportions of *Actinobacteria* and *Planctomycetes*, respectively. Medians of Simpson’s diversity indices were above 0.8 for all sample groups apart from the aquatic blank group (0.3), which had a high relative abundance of *Vibrionales* (i.e., above 15%) and was the only group with >1% of the extremophilic *Deinococcus–Thermus* phylum (i.e., almost 7%). The number of ASVs in each environment (only those that were >1% relative abundance are shown) was related to the number of samples from that environment, with there being the highest and lowest numbers of both ASVs and samples in the marine and terrestrial environments, respectively (Figs. 1 and 4), and each substratum type within each environment had ASVs that were unique (above 1% relative abundance in that treatment only) to it.

### Environmental variables—and not plastic type—have the largest impact on microbial composition

**Fig. 5:**
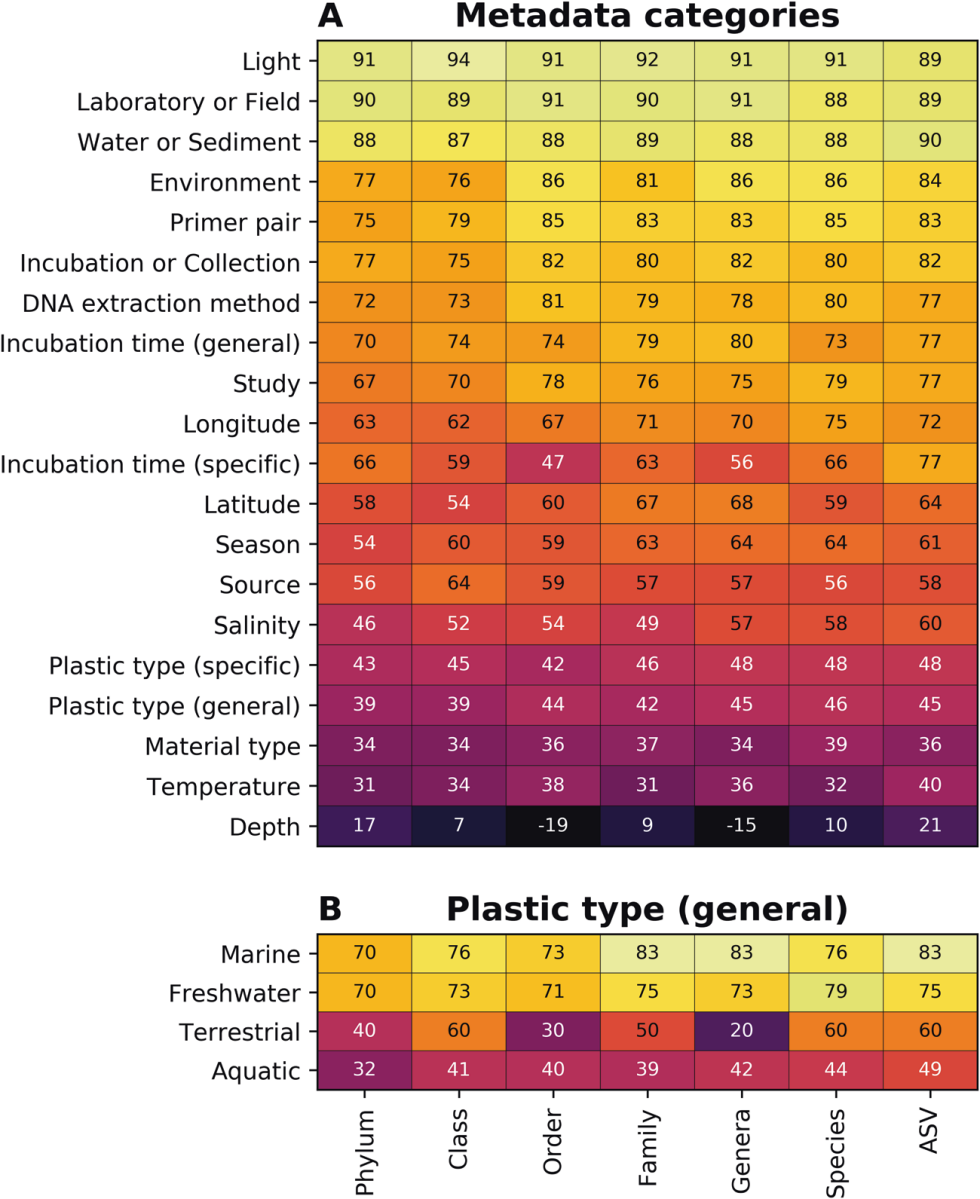
Classification accuracy for random forest models. Classification accuracy (%) for random forest models (classification or regression for discrete or continuous categories, respectively) constructed for (**A**) all samples grouped within different metadata categories or (**B**) samples within each environment grouped within plastic type (general) at different taxonomic levels. Random forest models are trained using a subset of 80% of samples (chosen randomly) and classification accuracy is based on testing using the remaining 20% of samples. Figure S4 shows the top most important features at the ASV level across all metadata categories while Supplementary Section 3 shows all taxonomic levels as well as metadata categories.

To determine which metadata categories have the largest impact on microbial composition, we constructed random forest models for all 20 categories—including factors such as environment, geographic location, temperature, primer pair, and plastic type to varying degrees of specificity—for each taxonomic level: phylum, class, order, family, genus, species, and ASV (i.e., 140 random forest models were constructed on the rarefied data). We found that models constructed using the microbial composition at the ASV and phylum levels were on average the most (67%) and least (61%) accurate, respectively, and that the light regime (whether samples were incubated at ambient or modified lighting conditions, e.g., shaded or a laboratory 16:8 light:dark cycle) was the metadata category with the highest classification accuracy (maximum 94% at the class level; Fig. 5A). The other metadata categories that were successful more than 80% of the time were: (i) whether the samples came from experiments carried out in the laboratory or the field (maximum accuracy 91% at order or genera level); (ii) whether the samples were incubated/collected from sediment or the water column (maximum accuracy 90% at the ASV level); (iii) the environment that the sample came from (maximum accuracy 86% at order, genera or species level); (iv) the primer pair used for sequencing (maximum accuracy 85% at order or species levels); (v) whether the sample was collected (i.e., unknown environmental residence time) or incubated for a known length of time (maximum accuracy 82% at order, genera or ASV level); and (vi) the DNA extraction method used (maximum accuracy 81% at order level). Depth consistently produced the models with the lowest classification accuracy (maximum accuracy 21% at the ASV level), with temperature also performing poorly (maximum accuracy 40% at the ASV level) and all other categories, such as plastic type, specific incubation time, and geographic location, having intermediate accuracy levels.

When we examine the taxa with the highest mean feature importance values (proportion that the classification accuracy decreases without that feature), we find that at all taxonomic levels besides ASV, it is a member of the *Bacteroidetes* with the highest values (maximum 0.159 at the phylum level; Fig. S4 and Supplementary Section 3); even though the *Bacteroidetes* are substantially less abundant than the *Proteobacteria* in all but the terrestrial environment (Fig. 4). The next highest feature importance values are generally from the *Proteobacteria*, and in particular the *Alphaproteobacteria* (e.g., the ASV with the highest mean value, ASV1197 *Erythrobacter*, 0.03), although the latitude and longitude usually have the highest individual importance values for both taxonomic groups. There are several metadata categories for which the taxa with high—or low—feature importance values are strongly correlated, for example: latitude and longitude (e.g., ASV1954 *Lentimonas* and ASV1197 *Erythrobacter*); study, primer pair, and DNA extraction method (e.g., ASV0715 AEGEAN-169 marine group and ASV0496 SAR116 clade); and source, material type, plastic type and whether the sample comes from an incubation or collection experiment (e.g., ASV0808 *Sphingomonadaceae*, and ASV4333 *Alteromonas*; Fig. S4).

### The Plastisphere includes potential plastic biodegraders and potentially pathogenic species

To determine the taxa that were potentially specific to plastics, samples were grouped by the environment they originated from and random forest models were trained on general plastic type (as in Fig. 1D). Random forest classification models showed that splitting samples by environment improved the classification accuracy of the plastic type metadata category (to a maximum of 83% at the family, genus or ASV level in the marine environment) and the accuracy remained at below 50% at all taxonomic levels in only the aquatic environment (Fig. 5B and Supplementary Section 3). We were able to identify taxa that were significantly differentially abundant between substratum types in all environments, although in the terrestrial environment we only found significant differences at the genus level (and not at any other taxonomic level; Fig. S5 and Supplementary Section 3). In the marine environment, these included large numbers of *Alphaproteobacteria*, that were more abundant in planktonic samples, while the *Bacteroidetes* and *Gammaproteobacteria* were more abundant in biofilm samples (either plastics or controls). Of particular interest were the taxonomic groups contained within the hydrocarbonoclastic *Oceanospirillales* (the families *Saccharospirillaceae* and *Halomonadaceae* and genera *Alcanivorax* and *Oleiphilus*) and *Alteromonadales* (the families *Alteromonadaceae*, *Marinobacteraceae* and *Pseudoalteromonadaceae*) [118] orders that were more abundant in plastic samples than control biofilms, with the *Oceanospirillales* generally being more abundant in the aliphatic plastic samples and the *Alteromonadales* generally being more abundant in the other plastic samples (Fig. S5 and Supplementary Section 3). The *Cyanobacteria* and the hydrocarbonoclastic *Halomonadaceae* were always more abundant in the unknown plastic samples (Fig. S5 and Supplementary Section 3), which were collected from the ocean after unknown residence times (Supplementary Table S2).

In the aquatic environment, there were also several potentially hydrocarbonoclastic taxonomic groups that were more abundant in plastic than other samples (the families *Thalassospiraceae*, *Alteromonadaceae*, *Pseudoalteromonadaceae*, *Saccharospirillaceae* and *Xanthomonadaceae* and genera *Idiomarina* and *Alcanivorax*), however, this difference was only significant for the potentially PAH-degrading *Spirosomaceae* family [119]. In the freshwater environment, there were also several taxonomic groups that have either been isolated from hydrocarbon-contaminated environments (ASV0388 *Arcobacter* and ASV0394 *Arcobacter cryaerophilus* [120]; ASV3841 Unclassified *Xanthomonadaceae* [118]) or have been suggested to be capable of hydrocarbon (Unclassified *Immundisolibacteraceae* genera [121]) or biodegradable plastic (ASV2841 *Flavobacterium* [122]) degradation that were significantly more abundant in the unknown plastic samples. Those that were more abundant in other plastic samples, were classes (*Acidobacteriia* and *Thermoanaerobaculia*) and orders (OPB56—*Ignavibacteria*—*Caulobacterales*, PB19—*Deltaproteobacteria*—and *Verrucomicrobiales*), and it is therefore much more difficult to speculate on the metabolic potential of these taxa. The same was true of the terrestrial environment, where many of the genera that were more abundant in the plastic samples remained unclassified at the order level (e.g., *Frankiales* and *Microtrichales*), although several genera known to be able to degrade hydrocarbons were also identified, e.g., *Arthrobacter*, *Acinetobacter* [118], *Methylocaldum* [123], and *Nitrosomonas* [124] as being significantly more abundant in the plastic (unknown plastics only) than control biofilm samples.

Previous studies have indicated the presence of potentially pathogenic hitchhikers in the Plastisphere, e.g., [30, 64, 125–128] however, other studies have found that potentially pathogenic species were actually higher in abundance on natural substrates, such as wood, e.g., [25]. Therefore, we investigated taxa that could potentially be pathogenic and that were more abundant on plastics than other samples. We found several taxonomic groups that: (i) were potential pathogens of animals—*Tenacibaculum* (abundant in marine aliphatic plastic samples) and unclassified *Pirellulaceae* (abundant in marine other plastic samples); (ii) contained lower taxonomic levels with human pathogens—*Clostridiales* and ASV0589 *Thalassospira* (both abundant in marine other plastic samples) and *Chlamydiae* (abundant in marine unknown plastic samples). However, the ability of a bacterium to be pathogenic is likely dependent on the presence of specific virulence factors which are often in mobile genetic elements [129]. The resolution of amplicon sequencing data is therefore insufficient to determine pathogenesis. There were also taxa, for example the genus *Vibrio*, of which members are potentially capable of degrading plastics [130] as well as being pathogens of humans and other organisms [129]. Curiously, we found that the *Vibrionales* were more abundant in plastic samples than control biofilm or planktonic samples in the marine environment, but in the aquatic environment they were more abundant in planktonic than any other samples. This highlights the need for other methods that will enable differentiation between pathogens and non-pathogens, as well as between strains that are capable of plastic degradation and those that are not (as discussed in detail in [131]).

### Microbial community succession on different material types

**Fig. 6:**
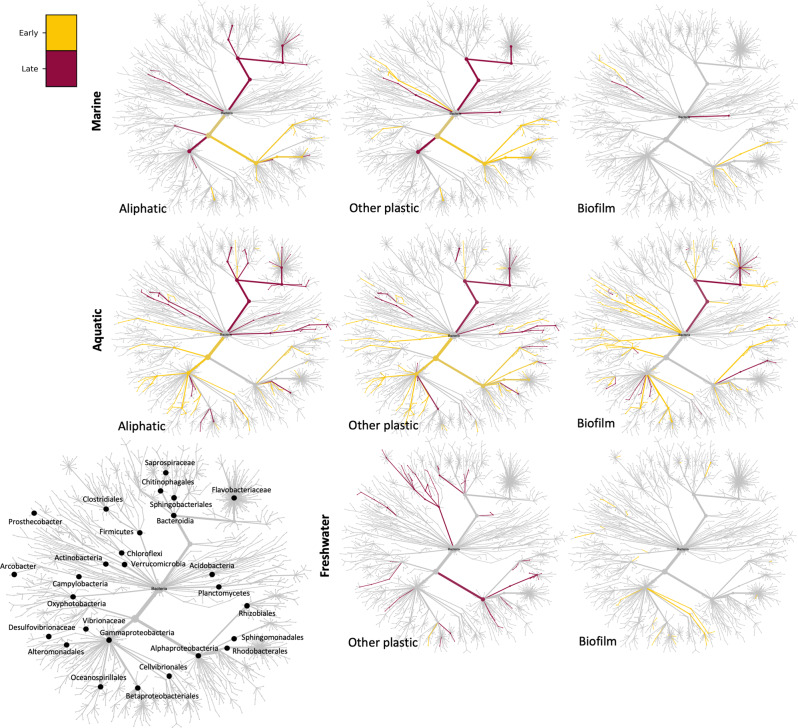
Differential abundance of taxa between early and late incubation times. Heat trees showing differential abundance of taxa between early and late incubation times (up to or above 7 days, respectively) within substratum types (aliphatic, other plastic, and control biofilms) for each of the marine, aquatic and freshwater environments where samples were taken at different time points. Strength of colors indicates differential abundance, with gray indicating no significant difference (*p* > 0.05; Wilcoxon rank sum tests with holm-bonferroni false discovery rate correction), and strong yellow or red colors indicating that log_2_ fold change is at least threefold higher in the early or late samples, respectively. Some key taxa are indicated in the empty, larger tree on the left and all are shown in Supplementary Section 4. Tests between different substrata at the same time point as well as between different substrata at all time points are also shown in Supplementary Sections 4 and 5. All terrestrial samples were collected after an unknown environmental residence time and could therefore not be included.

When we examined the taxa that discriminate between early and late incubation times (up to or above 7 days of incubation, respectively), we find some fairly consistent patterns across the marine and aquatic environments: (i) the *Bacteroidetes* were always significantly more abundant at later time points (where they differed in abundance between early and late time points); and (ii) the *Alphaproteobacteria* (or orders within the *Alphaproteobacteria*) are always more abundant at earlier time points (Fig. 6). Other taxonomic groups were more dependent upon the specific comparison. For example, the *Gammaproteobacteria*, that were more abundant at later time points in marine plastic samples (aliphatic and other plastics), but were generally more abundant at early time points in the aquatic environment, with the hydrocarbonoclastic *Oceanospirillales* and *Alteromonadales* in particular being more abundant at early time points. There were also a large number of phyla that were significantly (*p* < 0.05; Wilcoxon rank sum tests with holm-bonferroni false discovery rate correction) more abundant at early than late time points in the aquatic control biofilm samples, namely the *Chloroflexi*, *Verrucomicrobia*, *Actinobacteria*, *Cyanobacteria*, and *Deltaproteobacteria* (Fig. 6). Both other plastic and control biofilm samples in the freshwater environment showed different successional patterns than in either the marine or aquatic environments, although some members of the *Bacteroidia* were still more abundant at later time points. In the plastic samples, only the *Methylophilaceae* were more abundant at early than late incubation stages, while in the control biofilm samples there were no taxa that were significantly more abundant at late incubation stages.

The differences between substratum types were also compared at different time points, revealing different patterns between the marine and aquatic environments; there were more differences between sample pairings at late time points in the marine and at early time points in the aquatic environment, with a greater number of differences overall in the aquatic environment (Supplementary Sections 4 and 5). These again included the hydrocarbonoclastic *Oceanospirillales* and *Alteromonadales*, that were more abundant in aliphatic plastic samples than control biofilms at early time points, but these differences had largely disappeared by later time points. In the freshwater environment there were not enough samples for comparisons to be made for most sample pairings (only the other plastic and control biofilm samples had both early and late time points), however, differences between sample types were only observed at late incubation times (between other and unknown plastics as well as other plastics and control biofilms).

## Discussion

The re-analysis of 35 Plastisphere amplicon sequencing studies that we present here has allowed for a comprehensive characterization of the 16S rRNA gene community that colonizes plastics in marine, freshwater, other aquatic and terrestrial environments. We trained random forest models to classify samples within metadata categories, revealing that, overall, there were a number of variables—both relating to study design and environmental factors—that were better able to differentiate between sample groupings than plastic type (Fig. 5A). This agrees with the small re-analysis of studies presented by Oberbeckmann and Labrenz (2020; *n* = 5) [45], who reported that geographic location and salinity, but not substrate type, significantly discriminated between different samples. They did not, however, investigate different aspects of study design, such as environment, light regime or DNA extraction methods and only included studies that used the same primer pair, all of which are categories that we found to have a higher classification accuracy than location (latitude and longitude) or salinity (Fig. 5A). We tried to control for primer pair and DNA extraction technique by agglomerating sequences based on phylogenetic placement, which is known to reduce the variation between different 16S rRNA gene regions [50], although there are some taxa that will not be amplified by particular primer pairs, including the hydrocarbonoclastic groups targeted by many Plastisphere studies [132].

When we split our analyses to different environments (marine, aquatic, freshwater, and terrestrial), we find that plastic type could be used to train random forest models that were up to 83% accurate (Fig. 5B). We were, therefore, able to show that the differences identified by individual studies between microbial biofilms on control substrates and the Plastisphere were the same across the Plastisphere studies that we re-analysed here, regardless of the methodological differences between studies. For example, we find that the hydrocarbonoclastic *Oceanospirillales* and *Alteromonadales*, both previously suggested to be plastic biodegraders, e.g., [35, 73], were both important for differentiating between different substrate types in the random forest models (i.e., between aliphatic plastics, other plastics, control biofilms, and planktonic communities). They were also identified as significantly differentially abundant between these substrates. Furthermore, we find that the *Oceanospirillales* are higher in abundance in aliphatic plastics while the *Alteromonadales* are higher in abundance in the other plastic types. This is an observation that, to our knowledge, has not been previously acknowledged. Members of both of these orders, e.g., *Alcanivorax* sp. (*Oceanospirillales*) [35] and *Alteromonas* sp. (*Alteromonadales*) [133], have been reported to be able to degrade PE (aliphatic plastic) or PET (other plastic), respectively. However, these studies do not incorporate measurements of the plastic carbon assimilation into biomass (or of respiration) and therefore fall short of being definitive measurements of plastic biodegradation [134].

Almost 1,000 toxic chemicals are known to be associated with plastics [135]. These include manufacturing additives as well as weathering sub-products, most of which are not chemically bound and leach from plastics on environmental exposure [136]. They are more labile and are therefore more likely to be biodegraded by the Plastisphere than plastics themselves. However, there are only two studies included here that consider the effect that these have on community composition [31, 33]. For many plastics, these chemicals are not only additives but are also likely intermediates of both biotic and abiotic degradation. Cyclic and noncyclic dicarboxylic acids, for example, are the most commonly identified intermediates for the abiotic degradation of PE, PP, PS, and PET [137] and are also intermediates of biotic PET [138] and polyester [48] as well as plastic additive (plasticizer) [37] degradation. It has been suggested that the microbial community that is likely able to use these more labile chemicals is only present at earlier time points [31]. Almost all surfaces that enter the environment go through distinct and well-characterized stages of microbial community succession, and it is well known that microbial communities on different surfaces converge at later time points [139–141]. Here we have defined early incubation stages as before and later as after 1 week of incubation. However, due the relatively small number of studies that characterize the Plastisphere across a wide range of different incubation times, we do not actually know what early or late incubation stages are within the Plastisphere. Long residence times in the order of weeks to months have generally been favored by Plastisphere studies—the mean incubation time for all samples included here was 100 days—rather than the days that it typically takes for microbial communities to diverge from organisms that are efficient at degrading the surfaces they colonize to cheaters, cross-feeders, and grazers [139, 142]. This makes sense when we know that plastics may take longer than hundreds of years to be completely mineralized [143], but it also explains why several studies have found that potential degraders are only present at very low relative abundances [26] or are abundant only at early time points [31].

There are also several other areas that we have identified as needing further research before definitive conclusions may be made. For instance, there are currently only four studies performed in the terrestrial environment (and only two that could be included), all current Plastisphere studies were performed in the Northern Hemisphere and the majority were performed in temperate environments (Fig. 1). Whilst this hinders our ability to draw large-scale conclusions, it offers an opportunity for researchers to ensure that future data collected in these areas are comparable and address these knowledge gaps. We were unable to confirm or reject the suggestions that plastics carry higher abundances of pathogenic hitchhikers, e.g., [23, 30, 64, 126–128], and/or antimicrobial resistance genes, e.g., [39], than control biofilms. We do note, however, that due to their recalcitrance and buoyancy plastics present a higher chance of carrying pathogens or antimicrobial resistance genes across greater distances than most natural surfaces. We, along with many of the studies included here, have identified the presence of potential plastic biodegraders and potentially pathogenic organisms on plastics. However, the amplicon sequencing used does not usually give the resolution required to differentiate between closely related strains of the same species [144], for example between pathogenic and non-pathogenic *Vibrio* spp. [129, 145] or hydrocarbon degrading and nondegrading *Pseudomonas putida* (previously *P. oleovorans*) spp. [146]. The presence of genes encoding the production of enzymes used in hydrocarbon biodegradation [118], or specific virulence factors required for pathogenicity are often conferred by mobile genetic elements [129] and their presence is therefore not necessarily based in phylogeny. The use of metagenomics rather than amplicon sequencing in future studies would aid in determining whether these *potential* plastic biodegraders and *potential* pathogens are indeed biodegraders or pathogenic [131]. As discussed further in [131], it may even generate candidate enzymes that could be tested for their ability to degrade plastics, such as in Danso et al. [147] for PET hydrolases.

This study presents, for the first time, a comprehensive re-analysis of all Plastisphere studies that utilize amplicon sequencing of the 16S rRNA gene. We have revealed through machine learning methods that environmental factors, such as environment and light availability as well as aspects of study design, such as primer pair and incubation time play a large role in shaping Plastisphere community composition. Notably, we have identified members of the microbial community that are consistently more abundant in biofilms formed on plastics than control biofilms across multiple studies and environments. This highlights the urgent need to determine whether these microbes are capable of plastic biodegradation or are pathogens of humans or other organisms. We have also identified a number of other key areas in which we are lacking even basic knowledge and where future research should be directed. It is clear that plastic pollution is a key indicator of the Anthropocene and we must focus future research on gaps in our knowledge that we highlight here if we wish to mitigate its effects.

## Supplementary information

Supplementary Information

Supplementary Table S1

Supplementary Table S2

## Data Availability

All sequencing data used here were either obtained from publicly accessible databases or directly from the authors of the studies (full details are in Table S1B) and all scripts used during these analyses can be found in File S1 and at https://github.com/R-Wright-1/Plastisphere-MetaAnalysis. This includes a pipeline with step-by-step instructions and code for reproducing the analyses carried out here, with or without the inclusion of additional studies. All files used and produced during these analyses (e.g., QIIME2 objects, random forest output files and figures) are available on Figshare: raw read files in QIIME2 zipped format, 10.6084/m9.figshare.12931372; QIIME2 merged input files, including processed individual study and all merged study reads, 10.6084/m9.figshare.12217682; QIIME2 intermediate and output files 10.6084/m9.figshare.12227522; and all files created during the analysis 10.6084/m9.figshare.12227303.

## References

[1] Geyer R, Jambeck JR, Law KL (2017). Production, use, and fate of all plastics ever made. Sci Adv.

[2] Carr A (1987). Impact of nondegradable marine debris on the ecology and survival outlook of sea turtles. Mar Pollut Bull.

[3] Gregory MR (2009). Environmental implications of plastic debris in marine settings-entanglement, ingestion, smothering, hangers-on, hitch-hiking and alien invasions. Philos Trans R Soc.

[4] Browne MA, Dissanayake A, Galloway TS, Lowe DM, Thompson RC (2008). Ingested microscopic plastic translocates to the circulatory system of the mussel, Mytilus edulis (L.). Environ Sci Technol.

[5] Colabuono FI, Taniguchi S, Montone RC (2010). Polychlorinated biphenyls and organochlorine pesticides in plastics ingested by seabirds. Mar Pollut Bull.

[6] Ryan PG, Connell AD, Gardner BD (1988). Plastic ingestion and PCBs in seabirds: is there a relationship?. Mar Pollut Bull.

[7] Vered G, Kaplan A, Avisar D, Shenkar N (2019). Using solitary ascidians to assess microplastic and phthalate plasticizers pollution among marine biota: a case study of the Eastern Mediterranean and Red Sea. Mar Pollut Bull.

[8] Karbalaei S, Golieskardi A, Hamzah HB, Abdulwahid S, Hanachi P, Walker TR (2019). Abundance and characteristics of microplastics in commercial marine fish from Malaysia. Mar Pollut Bull.

[9] Rochman CM, Hoh E, Kurobe T, Teh SJ (2013). Ingested plastic transfers hazardous chemicals to fish and induces hepatic stress. Sci Rep.

[10] Teuten EL, Saquing JM, Knappe DRU, Barlaz MA, Jonsson S, Bjorn A (2009). Transport and release of chemicals from plastics to the environment and to wildlife. Philos Trans R Soc B Biol Sci.

[11] de Souza Machado AA, Kloas W, Zarfl C, Hempel S, Rillig MC (2018). Microplastics as an emerging threat to terrestrial ecosystems. Glob Chang Biol.

[12] Allen S, Allen D, Phoenix VR, Le Roux G, Durántez Jiménez P, Simonneau A (2019). Atmospheric transport and deposition of microplastics in a remote mountain catchment. Nat Geosci.

[13] Bergmann M, Mützel S, Primpke S, Tekman MB, Trachsel J, Gerdts G (2019). White and wonderful? Microplastics prevail in snow from the Alps to the Arctic. Sci Adv.

[14] Horton AA, Dixon SJ (2018). Microplastics: an introduction to environmental transport processes. Wiley Interdiscip Rev Water.

[15] Van Sebille E, Wilcox C, Sherman P, Hardesty BD, Law KL. Modelling global distribution, risk and mitigation strategies of floating plastic pollution. Geophys Research Abstract. EGU General Assembly. Vienna, Austria. 2016.

[16] Bakir A, Rowland SJ, Thompson RC (2012). Competitive sorption of persistent organic pollutants onto microplastics in the marine environment. Mar Pollut Bull.

[17] Andrady AL (2017). The plastic in microplastics: a review. Mar Pollut Bull.

[18] Amaral-Zettler LA, Zettler ER, Mincer TJ (2020). Ecology of the plastisphere. Nat Rev Microbiol.

[19] Derraik JGB (2002). The pollution of the marine environment by plastic debris: a review. Mar Pollut Bull.

[20] Gregory MR (1978). Accumulation and distribution of virgin plastic granules on New Zealand beaches. N Zeal J Mar Freshw Res.

[21] Barnes DKA (2002). Invasions by marine life on plastic debris. Nature.

[22] Darby RT, Kaplan AM (1968). Fungal susceptibility of polyurethanes. Appl Microbiol.

[23] Zettler ER, Mincer TJ, Amaral-Zettler LA (2013). Life in the “Plastisphere”: Microbial communities on plastic marine debris. Environ Sci Technol.

[24] Harrison JP, Sapp M, Schratzberger M, Osborn AM (2011). Interactions between microorganisms and marine microplastics: a call for research. Mar Technol Soc J.

[25] Kesy K, Oberbeckmann S, Kreikemeyer B, Labrenz M (2019). Spatial environmental heterogeneity determines young biofilm assemblages on microplastics in Baltic Sea Mesocosms. Front Microbiol.

[26] Kirstein IV, Wichels A, Krohne G, Gerdts G (2018). Mature biofilm communities on synthetic polymers in seawater - Specific or general?. Mar Environ Res.

[27] Kirstein IV, Wichels A, Gullans E, Krohne G, Gerdts G (2019). The plastisphere – Uncovering tightly attached plastic “specific” microorganisms. PLoS One.

[28] Oberbeckmann S, Osborn AM, Duhaime MB (2016). Microbes on a bottle: substrate, season and geography influence community composition of microbes colonizing marine plastic debris. PLoS One.

[29] Muthukrishnan T, Khaburi MAL, Abed RMM (2018). Fouling microbial communities on plastics compared with wood and steel: Are they substrate- or location-specific?. Micro Ecol.

[30] Oberbeckmann S, Kreikemeyer B, Labrenz M (2018). Environmental factors support the formation of specific bacterial assemblages on microplastics. Front Microbiol.

[31] Erni-Cassola G, Wright RJ, Gibson MI, Christie-Oleza JA (2019). Early colonization of weathered polyethylene by distinct bacteria in marine coastal seawater. Micro Ecol.

[32] Ogonowski M, Motiei A, Ininbergs K, Hell E, Gerdes Z, Udekwu KI (2018). Evidence for selective bacterial community structuring on microplastics. Environ Microbiol.

[33] Pinto M, Langer TM, Huffer T, Hofmann T, Herndl GJ, Hüffer T (2019). The composition of bacterial communities associated with plastic biofilms differs between different polymers and stages of biofilm succession. PLoS One.

[34] Kettner MT, Oberbeckmann S, Labrenz M, Grossart HP (2019). The eukaryotic life on microplastics in brackish ecosystems. Front Microbiol.

[35] Delacuvellerie A, Cyriaque V, Gobert S, Benali S, Wattiez R (2019). The plastisphere in marine ecosystem hosts potential specific microbial degraders including Alcanivorax borkumensis as a key player for the low-density polyethylene degradation. J Hazard Mater.

[36] Devi RS, Ramya R, Kannan K, Antony AR, Kannan VR (2019). Investigation of biodegradation potentials of high density polyethylene degrading marine bacteria isolated from the coastal regions of Tamil Nadu, India. Mar Pollut Bull.

[37] Wright RJ, Bosch R, Gibson MI, Christie-Oleza JA (2020). Plasticizer degradation by marine bacterial isolates: a proteogenomic and metabolomic characterization. Environ Sci Technol.

[38] De Tender CA, Devriese LI, Haegeman A, Maes S, Ruttink T, Dawyndt P (2015). Bacterial community profiling of plastic litter in the Belgian part of the North Sea. Environ Sci Technol.

[39] Laganà P, Caruso G, Corsi I, Bergami E, Venuti V, Majolino D (2019). Do plastics serve as a possible vector for the spread of antibiotic resistance? First insights from bacteria associated to a polystyrene piece from King George Island (Antarctica). Int J Hyg Environ Health.

[40] Yang Y, Liu G, Song W, Ye C, Lin H, Li Z (2019). Plastics in the marine environment are reservoirs for antibiotic and metal resistance genes. Environ Int.

[41] Zhang Y, Lu J, Wu J, Wang J, Luo Y (2020). Potential risks of microplastics combined with superbugs: Enrichment of antibiotic resistant bacteria on the surface of microplastics in mariculture system. Ecotoxicol Environ Saf.

[42] Wang S, Xue N, Li W, Zhang D, Pan X, Luo Y. Selectively enrichment of antibiotics and ARGs by microplastics in river, estuary and marine waters. Science Total Environment. 2020;708:134594. 10.1016/j.scitotenv.2019.134594 31796269

[43] Jacquin J, Cheng J, Odobel C, Pandin C, Conan P, Pujo-Pay M (2019). Microbial ecotoxicology of marine plastic debris: a review on colonization and biodegradation by the “Plastisphere”. Front Microbiol.

[44] Roager L, Sonnenschein EC (2019). Bacterial candidates for colonization and degradation of marine plastic debris. Environ Sci Technol.

[45] Oberbeckmann S, Labrenz M (2020). Marine microbial assemblages on microplastics: diversity, adaptation, and role in degradation. Ann Rev Mar Sci.

[46] Rogers KL, Carreres-calabuig JA, Gorokhova E, Posth NR. Micro-by-micro interactions: how microorganisms influence the fate of marine microplastics. Limnol Oceanogr Lett. 2020;5:1–19.

[47] Wei R, Zimmermann W (2017). Microbial enzymes for the recycling of recalcitrant petroleum-based plastics: how far are we?. Micro Biotechnol.

[48] Danso D, Chow J, Streit WR. Plastics: microbial degradation, environmental and biotechnological perspectives. Appl Environ Microbiol. 2019;85:1–14. 10.1128/AEM.01095-19 PMC6752018 31324632

[49] Salvador de Lara M, Abdulmutalib U, Gonzalez J, Kim J, Smith AA, Faulon J-L (2019). Genes for a circular and sustainable bio-PET economy. Genes (Basel).

[50] Janssen S, Mcdonald D, Gonzalez A, Navas-molina JA, Jiang L, Xu Z (2018). Phylogenetic placement of exact amplicon sequences improves associations with clinical information. mSystems.

[51] Abed RMM, Muthukrishnan T, Al Khaburi M, Al-Senafi F, Munam A, Mahmoud H (2020). Degradability and biofouling of oxo-biodegradable polyethylene in the planktonic and benthic zones of the Arabian Gulf. Mar Pollut Bull.

[52] Amaral-Zettler LA, Zettler ER, Slikas B, Boyd GD, Melvin DW, Morrall CE (2015). The biogeography of the Plastisphere: implications for policy. Front Ecol Environ.

[53] Arias-Andres M, Klümper U, Rojas-Jimenez K, Grossart H-P (2018). Microplastic pollution increases gene exchange in aquatic ecosystems. Environ Pollut.

[54] Bakal T, Janata J, Sabova L, Grabic R, Zlabek V, Najmanova L (2019). Suitability and setup of next-generation sequencing-based method for taxonomic characterization of aquatic microbial biofilm. Folia Microbiol.

[55] Canada P, Pereira A, Nogueira N, Png-Gonzalez L, Andrade C, Xavier R (2020). Analysis of bacterial microbiome associated with nylon and copper nets in an aquaculture context. Aquaculture.

[56] Curren E, Leong SCY (2019). Profiles of bacterial assemblages from microplastics of tropical coastal environments. Sci Total Environ.

[57] Dussud C, Hudec C, George M, Fabre P, Higgs P, Bruzaud S (2018). Colonization of non-biodegradable and biodegradable plastics by marine microorganisms. Front Microbiol.

[58] Dussud C, Meistertzheim AL, Conan P, Pujo-pay M, George M, Fabre P (2018). Evidence of niche partitioning among bacteria living on plastics, organic particles and surrounding seawaters. Environ Pollut.

[59] Frère L, Maignien L, Chalopin M, Huvet A, Rinnert E, Morrison H (2018). Microplastic bacterial communities in the bay of brest: influence of polymer type and size. Environ Pollut.

[60] Gong M, Yang G, Zhuang L, Zeng EY (2019). Microbial biofilm formation and community structure on low-density polyethylene microparticles in lake water microcosms. Environ Pollut.

[61] Hoellein T, Rojas M, Pink A, Gasior J, Kelly J (2014). Anthropogenic litter in urban freshwater ecosystems: distribution and microbial interactions. PLoS One.

[62] Hoellein TJ, McCormick AR, Hittie J, London MG, Scott JW, Kelly JJ (2017). Longitudinal patterns of microplastic concentration and bacterial assemblages in surface and benthic habitats of an urban river. Freshw Sci.

[63] Huang Y, Zhao Y, Wang J, Zhang M, Jia W, Qin X (2019). LDPE microplastic films alter microbial community composition and enzymatic activities in soil. Environ Pollut.

[64] Jiang P, Zhao S, Zhu L, Li D (2018). Microplastic-associated bacterial assemblages in the intertidal zone of the Yangtze Estuary. Sci Total Environ.

[65] McCormick A, Hoellein TJ, Mason SA, Schluep J, Kelly JJ (2014). Microplastic is an abundant and distinct microbial habitat in an Urban river. Environ Sci Technol.

[66] McCormick AR, Hoellein TJ, London MG, Hittie J, Scott JW, J. KJ. Microplastic in surface waters of urban rivers: concentration, sources, and associated bacterial assemblages. Ecosphere. 2016;7:1–22.

[67] Miao L, Wang P, Hou J, Yao Y, Liu Z, Liu S (2019). Distinct community structure and microbial functions of biofilms colonizing microplastics. Sci Total Environ.

[68] Parrish K, Fahrenfeld NL (2019). Microplastic biofilm in fresh- and wastewater as a function of microparticle type and size class. Environ Sci Water Res Technol.

[69] Pollet T, Berdjeb L, Garnier CC, Durrieu GG, Le Poupon C, Misson B (2018). Prokaryotic community successions and interactions in marine biofilms: the key role of Flavobacteriia. FEMS Micro Ecol.

[70] Puglisi E, Romaniello F, Galletti S, Boccaleri E, Frache A, Cocconcelli PS (2019). Selective bacterial colonization processes on polyethylene waste samples in an abandoned landfill site. Sci Rep.

[71] Rosato A, Barone M, Negroni A, Brigidi P, Fava F, Xu P (2019). Microbial colonization of different microplastic types and biotransformation of sorbed PCBs by a marine anaerobic bacterial community. Sci Total Environ.

[72] Syranidou E, Karkanorachaki K, Amorotti F, Repouskou E, Kroll K, Kolvenbach B (2017). Development of tailored indigenous marine consortia for the degradation of naturally weathered polyethylene films. PLoS One.

[73] Syranidou E, Karkanorachaki K, Amorotti F, Franchini M, Repouskou E, Kaliva M (2017). Biodegradation of weathered polystyrene films in seawater microcosms. Sci Rep.

[74] Syranidou E, Karkanorachaki K, Amorotti F, Avgeropoulos A, Kolvenbach B, Zhou N (2019). Biodegradation of mixture of plastic films by tailored marine consortia. J Hazard Mater.

[75] Tagg AS, Oberbeckmann S, Fischer D, Kreikemeyer B, Labrenz M (2019). Paint particles are a distinct and variable substrate for marine bacteria. Mar Pollut Bull.

[76] Woodall LC, Jungblut AD, Hopkins K, Id AH, Robinson F, Gwinnett C, et al. Deep-sea anthropogenic macrodebris harbours rich and diverse communities of bacteria and archaea. PLoS ONE. 2018;13:1–11. 10.1371/journal.pone.0206220 PMC6261660 30485275

[77] Wu N, Zhang Y, Zhao Z, He J, Li W, Li J, et al. Colonization characteristics of bacterial communities on microplastics compared with ambient environments (water and sediment) in Haihe Estuary. Sci Total Environ. 2019;708:134876. 10.1016/j.scitotenv.2019.134876 31740062

[78] Xu X, Wang S, Gao F, Li J, Zheng L, Sun C (2019). Marine microplastic-associated bacterial community succession in response to geography, exposure time, and plastic type in China’s coastal seawaters. Mar Pollut Bull.

[79] Zhang M, Zhao Y, Qin X, Jia W, Chai L, Huang M (2019). Microplastics from mulching film is a distinct habitat for bacteria in farmland soil. Sci Total Environ.

[80] Kettner MT, Rojas-Jimenez K, Oberbeckmann S, Labrenz M, Grossart H-P (2017). Microplastics alter composition of fungal communities in aquatic ecosystems. Environ Microbiol.

[81] Didier D, Anne M, Alexandra TH (2017). Plastics in the North Atlantic garbage patch: a boat-microbe for hitchhikers and plastic degraders. Sci Total Environ.

[82] Esan EO, Abbey, Lord, Yurgel S (2019). Exploring the long-term effect of plastic on compost microbiome. PLoS One.

[83] De Tender C, Devriese LI, Haegeman A, Maes S, Vangeyte J, Cattrijsse A (2017). Temporal dynamics of bacterial and fungal colonization on plastic debris in the north sea. Environ Sci Technol.

[84] Pinnell LJ, Turner JW (2019). Shotgun metagenomics reveals the benthic microbial community response to plastic and bioplastic in a coastal marine environment. Front Microbiol.

[85] Bryant JA, Clemente TM, Viviani DA, Fong AA, Thomas KA, Kemp P (2016). Diversity and activity of communities inhabiting plastic debris in the North Pacific Gyre. mSystems.

[86] Wu X, Pan J, Li M, Li Y, Bartlam M, Wang Y. Selective enrichment of bacterial pathogens by microplastic biofilm. Water Res. 2019;165:114979. 10.1016/j.watres.2019.114979 31445309

[87] Comeau AM, Douglas GM, Langille MGI (2017). Microbiome helper: a custom and streamlined workflow for microbiome research. mSystems.

[88] Bolyen E, Rideout JR, Dillon MR, Bokulich NA, Abnet CC, Al-Ghalith GA (2019). Reproducible, interactive, scalable and extensible microbiome data science using QIIME 2. Nat Biotechnol.

[89] Ewels P, Magnusson M, Lundin S, Käller M (2016). MultiQC: summarize analysis results for multiple tools and samples in a single report. Bioinformatics.

[90] Martin M (2011). Cutadapt removes adapter sequences from high-throughput sequencing reads. EMBnet J.

[91] Rognes T, Flouri T, Nichols B, Quince C, Mahé F (2016). VSEARCH: a versatile open source tool for metagenomics. PeerJ.

[92] Amir A, Daniel M, Navas-Molina J, Kopylova E, Morton J, Xu ZZ (2017). Deblur rapidly resolves single-nucleotide community sequence patterns. Am Soc Microbiol.

[93] Quast C, Pruesse E, Yilmaz P, Gerken J, Schweer T, Glo FO (2013). The SILVA ribosomal RNA gene database project: improved data processing and web-based tools. Nucleic Acids Res.

[94] Cock PJA, Antao T, Chang JT, Chapman BA, Cox CJ, Dalke A (2009). Biopython: freely available Python tools for computational molecular biology and bioinformatics. Bioinformatics.

[95] Harrell FE, Lee KL, Mark DB (2005). Multivariable prognostic models: issues in developing models, evaluating assumptions and adequacy, and measuring and reducing errors. Stat Med.

[96] Hunter JD (2007). Matplotlib: a 2D graphics environment. Comput Sci Eng.

[97] McKinney W. Data structures for statistical computing in Python. In: van der Walt S, Millman J, editors. Proc. 9th Python Sci. Conf. 2010.

[98] Virtanen P, Gommers R, Oliphant TE, Haberland M, Reddy T, Cournapeau D (2020). SciPy 1.0: fundamental algorithms for scientific computing in Python. Nat Methods.

[99] Pedregosa F, Weiss R, Brucher M (2011). Scikit-learn: machine learning in Python. J Mach Learn Res.

[100] Paradis E, Schliep K (2019). Ape 5.0: an environment for modern phylogenetics and evolutionary analyses in R. Bioinformatics.

[101] R Core Team. R: A language and environment for statistical computing. R Foundation for Statistical Computing, Vienna, Austria. 2013. http://www.R-project.org/.

[102] Hadley W, François R, Henry L, Müller K. dplyr: a grammar of data manipulation. 2020. https://dplyr.tidyverse.org/.

[103] Hothorn T, Hornik K. exactRankTests: exact distributions for rank and permutation tests. 2019. https://rdrr.io/cran/exactRankTests/.

[104] Campitelli E. ggnewscale: multiple fill and colour scales in ‘ggplot2’. https://rdrr.io/cran/ggnewscale/.

[105] Wickham H (2016). ggplot2: elegant graphics for data analysis.

[106] Yu G, Smith DK, Zhu H, Guan Y, Lam TT (2017). GGTREE: an R package for visualization and annotation of phylogenetic trees with their covariates and other associated data. Methods Ecol Evol.

[107] Xie Y. Knitr: a comprehensive tool for reproducible research in R. In: Stodden V, Leisch F, Peng RD, editors. Implementing reproducible research. New York: Taylor and Francis; 2014.

[108] Foster ZSL, Sharpton TJ, Grünwald NJ (2017). Metacoder: an R package for visualization and manipulation of community taxonomic diversity data. PLoS Comput Biol.

[109] Lahti L, Shetty S. Microbiome R package. 2019. https://microbiome.github.io/tutorials/.

[110] Pinheiro J, Bates D, DebRoy S, Sarkar D. nlme: linear and nonlinear mixed effects models. 2020. https://www.researchgate.net/publication/303803175_Nlme_Linear_and_Nonlinear_Mixed_Effects_Models.

[111] Silverman JD, Washburne AD, Mukherjee S, David LA (2017). A phylogenetic transform enhances analysis of compositional microbiota data. Elife.

[112] McMurdie PJ, Holmes S (2013). phyloseq: an R package for reproducible interactive analysis and graphics of microbiome census data. PLoS One.

[113] Ushey K, Allaire J, Tang Y. Reticulate: interface to ‘Python’. 2020.

[114] Dixon P (2003). VEGAN, a package of R functions for community ecology. J Veg Sci.

[115] Lozupone C, Knight R (2005). UniFrac: a new phylogenetic method for comparing microbial communities. Appl Environ Microbiol.

[116] Gloor GB, Macklaim JM, Pawlowsky-Glahn V, Egozcue JJ (2017). Microbiome datasets are compositional: and this is not optional. Front Microbiol.

[117] Weiss S, Xu ZZ, Peddada S, Amir A, Bittinger K, Gonzalez A (2017). Normalization and microbial differential abundance strategies depend upon data characteristics. Microbiome.

[118] Prince RC, Gramain A, McGenity TJ. Prokaryotic hydrocarbon degraders. In: Timmis K, editor. Handbook of hydrocarbon and lipid microbiology. Berlin, Heidelberg: Springer; 2010. p. 1669–92.

[119] Zhou HW, Luan TG, Zou F, Tam NFY (2008). Different bacterial groups for biodegradation of three- and four-ring PAHs isolated from a Hong Kong mangrove sediment. J Hazard Mater.

[120] Evans MV, Panescu J, Hanson AJ, Welch SA, Sheets JM, Nastasi N (2018). Members of marinobacter and arcobacter influence system biogeochemistry during early production of hydraulically fractured natural gas wells in the appalachian basin. Front Microbiol.

[121] Corteselli EM, Aitken MD, Singleton DR (2017). Description of Immundisolibacter cernigliae gen. Nov., sp. nov., a high-molecular-weight polycyclic aromatic hydrocarbondegrading bacterium within the class Gammaproteobacteria, and proposal of Immundisolibacterales ord. nov. and Immundisolibacteraceae fa. Int J Syst Evol Microbiol.

[122] Feng XM, Tan X, Jia L, Long PP, Han L, Lv J (2015). Flavobacterium buctense sp. nov., isolated from freshwater. Arch Microbiol.

[123] Rochman FF, Sheremet A, Tamas I, Saidi-Mehrabad A, Kim JJ, Dong X (2017). Benzene and naphthalene degrading bacterial communities in an oil sands tailings pond. Front Microbiol.

[124] John RC, Okpokwasili GC (2012). Crude oil-degradation and plasmid profile of nitrifying bacteria isolated from oil-impacted mangrove sediment in the Niger Delta of Nigeria. Bull Environ Contam Toxicol.

[125] Zettler ER, Mincer TJ, Amaral-Zettler LA (2013). Life in the ‘plastisphere’: microbial communities on plastic marine debris. Environ Sci Technol.

[126] Keswani A, Oliver DM, Gutierrez T, Quilliam RS (2016). Microbial hitchhikers on marine plastic debris: human exposure risks at bathing waters and beach environments. Mar Environ Res.

[127] Kirstein IV, Kirmizi S, Wichels A, Garin-Fernandez A, Erler R, Löder M (2016). Dangerous hitchhikers? Evidence for potentially pathogenic Vibrio spp. on microplastic particles. Mar Environ Res.

[128] Silva MM, Maldonado GC, Castro RO, de Sá Felizardo J, Cardoso RP, Anjos RMdos (2019). Dispersal of potentially pathogenic bacteria by plastic debris in Guanabara Bay, RJ, Brazil. Mar Pollut Bull.

[129] Sakib SN, Reddi G, Almagro-Moreno S (2018). Environmental role of pathogenic traits in Vibrio cholerae. J Bacteriol.

[130] Raghul SS, Bhat SG, Chandrasekaran M, Francis V, Thachil ET (2014). Biodegradation of polyvinyl alcohol-low linear density polyethylene-blended plastic film by consortium of marine benthic vibrios. Int J Environ Sci Technol.

[131] Wright RJ, Erni-Cassola G, Zadjelovic V, Latva M, Christie-Oleza J (2020). Marine plastic debris – a new surface for microbial colonization. Environ Sci Technol.

[132] Berry D, Gutierrez T, Waite DW (2017). Evaluating the detection of hydrocarbon-degrading bacteria in 16S rRNA gene sequencing surveys. Front Microbiol.

[133] Webb H. Biodegradation of poly(ethylene terephthalate) by marine bacteria, and strategies for its enhancement. Swinburne University of Technology: Melbourne, Australia. 2012.

[134] Zumstein MT, Narayan R, Kohler H-PE, McNeill K, Sander M (2019). Dos and do nots when assessing the biodegradation of plastics. Environ Sci Technol.

[135] Groh KJ, Backhaus T, Carney-Almroth B, Geueke B, Inostroza PA, Lennquist A (2019). Overview of known plastic packaging-associated chemicals and their hazards. Sci Total Environ.

[136] Hahladakis JN, Velis CA, Weber R, Iacovidou E, Purnell P (2018). An overview of chemical additives present in plastics: migration, release, fate and environmental impact during their use, disposal and recycling. J Hazard Mater.

[137] Gewert B, Plassmann M, Sandblom O, Macleod M (2018). Identification of chain scission products released to water by plastic exposed to ultraviolet light. Environ Sci Technol Lett.

[138] Yoshida S, Hiraga K, Takehana T, Taniguchi I, Yanaji H, Maeda Y (2016). A bacterium that degrades and assimilates poly(ethyleneterephthalate). Science.

[139] Datta MS, Sliwerska E, Gore J, Polz MF, Cordero OX (2016). Microbial interactions lead to rapid micro-scale successions on model marine particles. Nat Commun.

[140] Dang H, Li T, Chen M, Huang G (2008). Cross-ocean distribution of Rhodobacterales bacteria as primary surface colonizers in temperate coastal marine waters. Appl Environ Microbiol.

[141] Wu X, Wu L, Liu Y, Zhang P, Li Q, Zhou J (2018). Microbial interactions with dissolved organic matter drive carbon dynamics and community succession. Front Microbiol.

[142] Wright RJ, Gibson MI, Christie-Oleza JA (2019). Understanding microbial community dynamics to improve optimal microbiome selection. Microbiome.

[143] Andrady AL. Persistence of plastic litter in the oceans. In: Bergmann M, Gutow L, Klages M, editors. Marine Anthropogenic Litter. Springer Open: Springer Nature Switzerland. 2015. p. 57–72.

[144] Johnson JS, Spakowicz DJ, Hong BY, Petersen LM, Demkowicz P, Chen L (2019). Evaluation of 16S rRNA gene sequencing for species and strain-level microbiome analysis. Nat Commun.

[145] Horseman MA, Surani S (2011). A comprehensive review of Vibrio vulnificus: an important cause of severe sepsis and skin and soft-tissue infection. Int J Infect Dis.

[146] Van Hamme JD, Singh A, Ward OP (2003). Recent advances in petroleum microbiology. Microbiol Mol Biol Rev.

[147] Danso D, Zimmermann, Schmeisser C, Chow J, Zimmermann W, Wei R (2018). New insights into the function and global distribution of polyethylene terephthalate (PET) degrading bacteria and enzymes in marine and terrestrial metagenomes. Appl Environ Microbiol.

