# Supplementary material for: Food or just a free ride? A meta-analysis reveals the global diversity of the Plastisphere
Source: ISME J. 2020 Nov 2;15(3):789–806. doi: 10.1038/s41396-020-00814-9 (PMC8027867; doi:10.1038/s41396-020-00814-9)
Supplement: Supplementary file 1 — Supplementary Information [file 41396_2020_814_MOESM1_ESM.pdf]

Supplementary Information for: Food or just a free ride? A meta-analysis reveals the global diversity of the Plastisphere

Robyn J. Wright [1,2*], Morgan G.I. Langille [2,3], Tony R. Walker [1]

[1] School for Resource and Environmental Studies, Dalhousie University, Halifax, Canada
[2] Department of Pharmacology, Faculty of Medicine, Dalhousie University, Canada
[3] Department of Microbiology and Immunology, Dalhousie University, Canada

* Corresponding author: Robyn J. Wright, robyn.wright@dal.ca

Supplementary figures

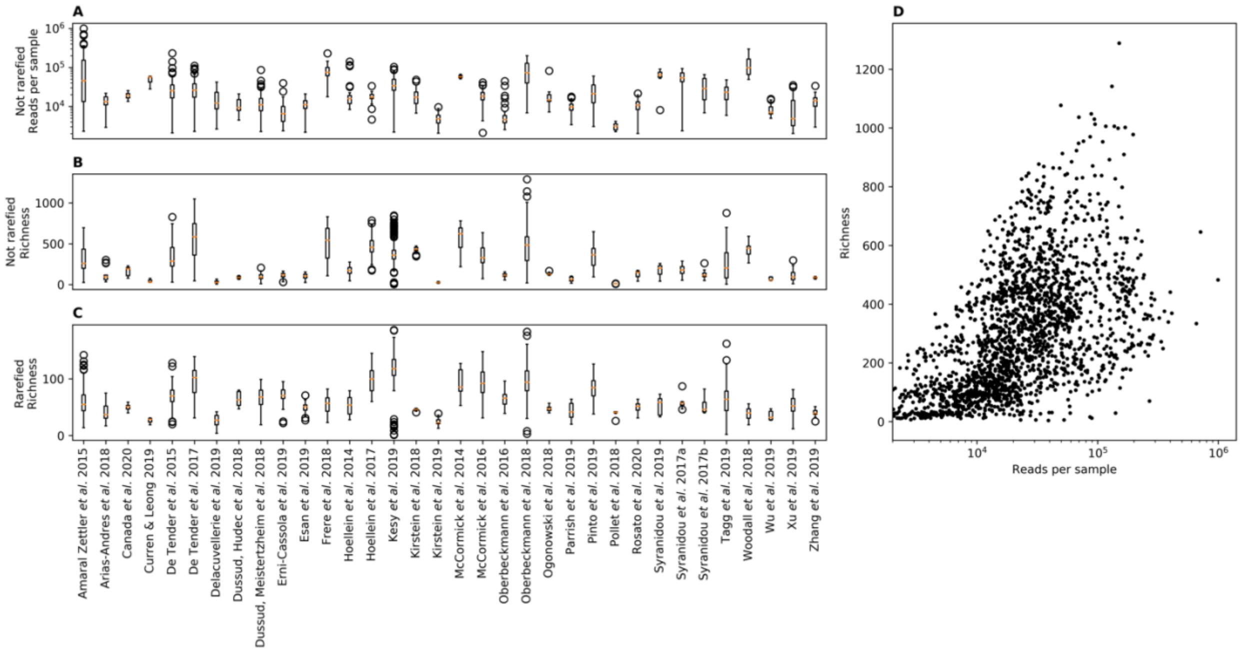

Figure S1. Sequencing depth (reads per sample; A) and richness (number of taxa) per sample before and after rarefaction to 2 000 sequences (B-C). Boxes show individual studies, with the upper and lower bounds of the box representing the upper and lower quartiles, orange lines representing the medians, error bars the confidence interval and individual points representing outliers. (D) shows a scatter plot of the number of reads and richness (before rarefaction), where each sample is represented as a separate point.

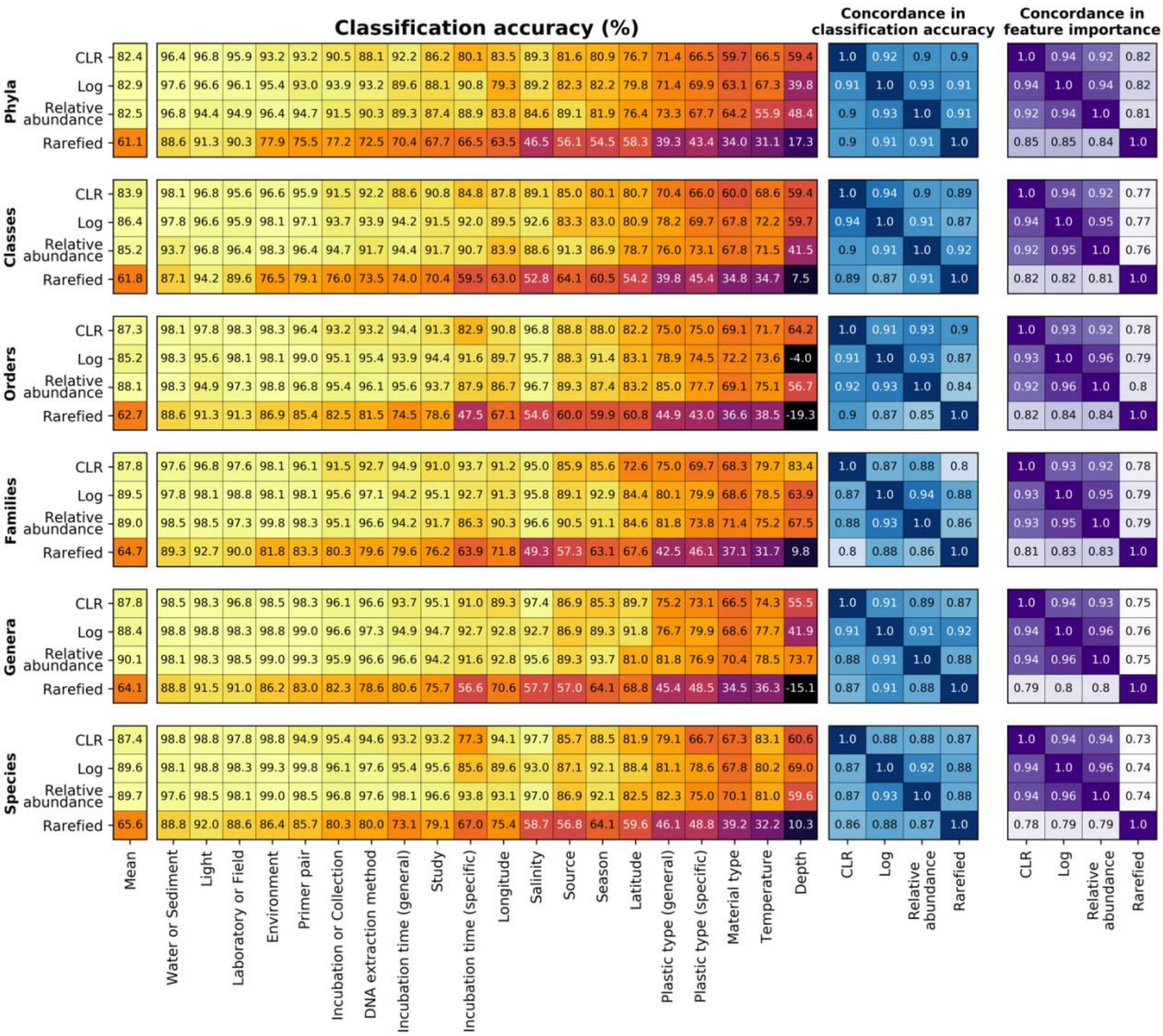

Figure S2. Classification accuracy (%) and concordance between classification accuracy and feature importance values for random forest models constructed for all metadata categories using data normalised in four different ways: (i) samples were converted to centered log ratios (with a pseudo count of half of the minimum non-zero count) (CLR); (ii) samples were transformed to log counts (with a pseudo count of 1) (Log); (iii) samples were converted to relative abundance (Relative abundance); and (iv) samples were rarefied and converted to relative abundance (Rarefied). More details on the differences between the random forest models generated using the different normalisation methods are in Supplementary Section 2.

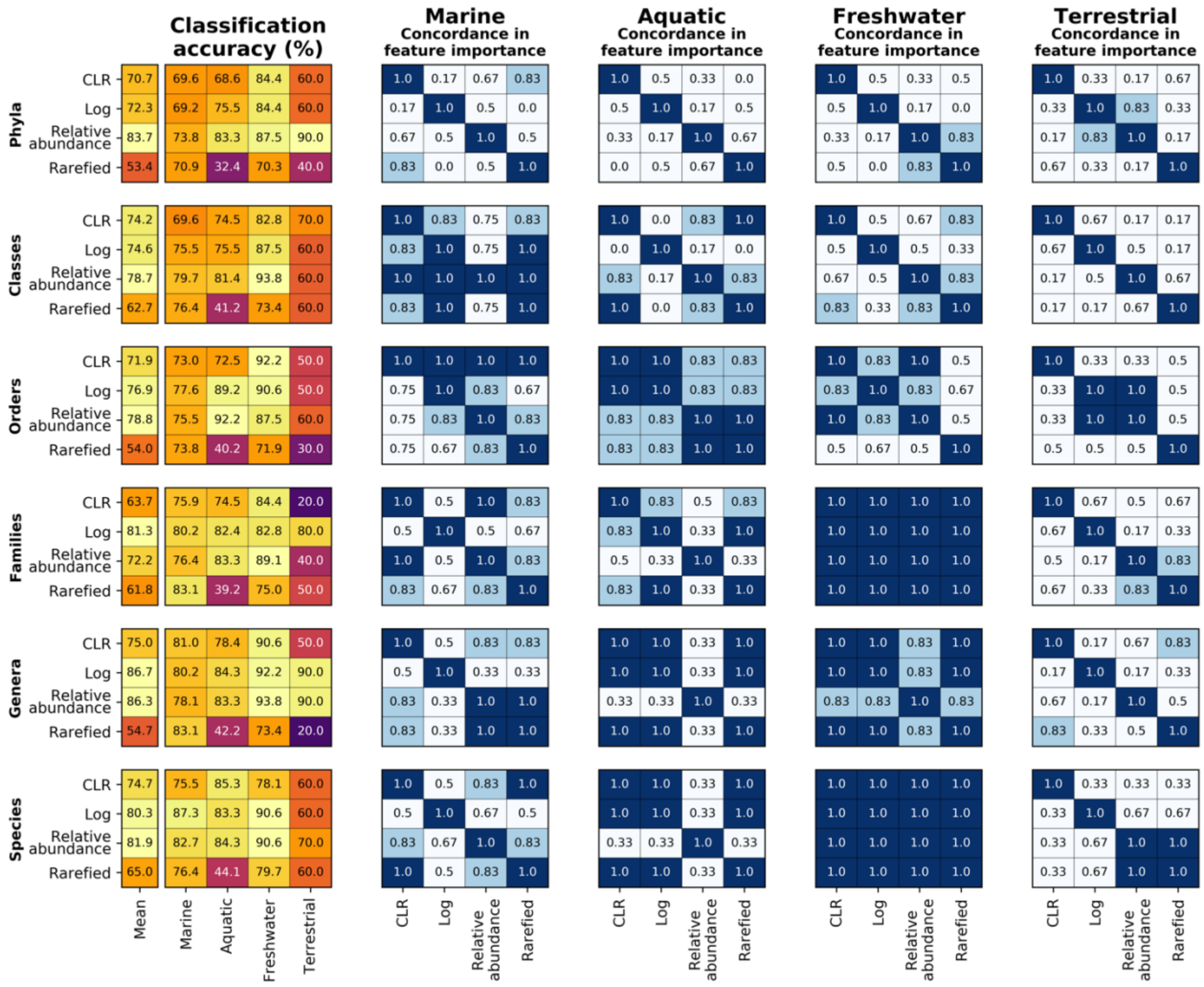

Figure S3 Classification accuracy (%) and concordance between feature importance values for random forest models constructed for general plastic type separated by environment using data normalised in four different ways: (i) samples were converted to centered log ratios (with a pseudo count of half of the minimum non-zero count) (CLR); (ii) samples were transformed to log counts (with a pseudo count of 1) (Log); (iii) samples were converted to relative abundance (Relative abundance); and (iv) samples were rarefied and converted to relative abundance (Rarefied). More details on the differences between the random forest models generated using the different normalisation methods are in Supplementary Section 2.

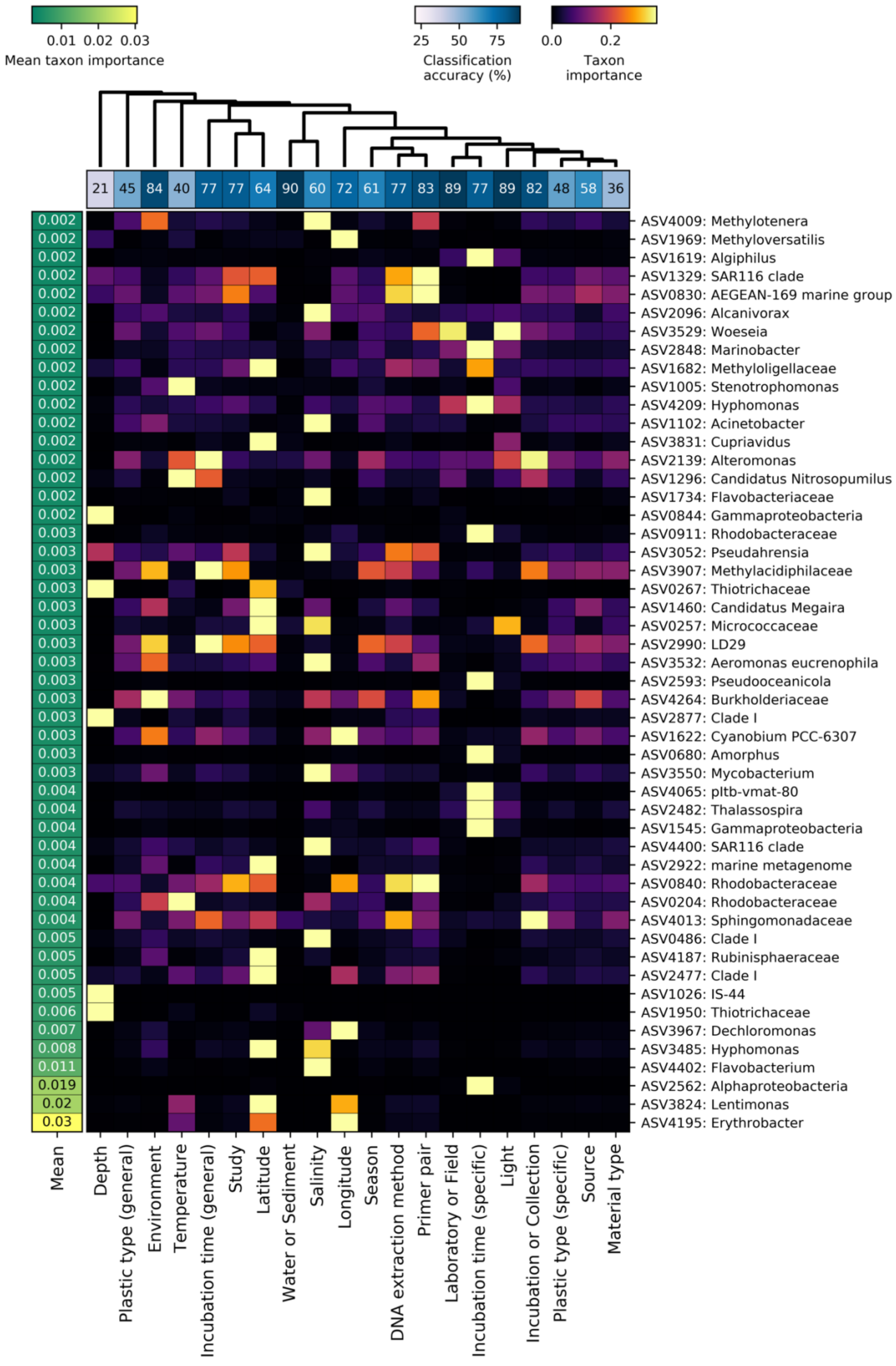

Mean taxon importance
0.01 0.02 0.03
Classification accuracy (%)
25 50 75
Taxon importance
0.0 0.2
21 45 84 40 77 77 64 90 60 72 61 77 83 89 77 89 82 48 58 36
Mean
Depth
Plastic type (general)
Environment
Temperature
Incubation time (general)
Study
Latitude
Water or Sediment
Salinity
Longitude
Season
DNA extraction method
Primer pair
Laboratory or Field
Incubation time (specific)
Light
Incubation or Collection
Plastic type (specific)
Source
Material type
ASV4009: Methylotenera
ASV1969: Methyloversatilis
ASV1619: Algiphilus
ASV1329: SAR116 clade
ASV0830: AEGEAN-169 marine group
ASV2096: Alcanivorax
ASV3529: Woeseia
ASV2848: Marinobacter
ASV1682: Methyloligellaceae
ASV1005: Stenotrophomonas
ASV4209: Hyphomonas
ASV1102: Acinetobacter
ASV3831: Cupriavidus
ASV2139: Alteromonas
ASV1296: Candidatus Nitrosopumilus
ASV1734: Flavobacteriaceae
ASV0844: Gammaproteobacteria
ASV0911: Rhodobacteraceae
ASV3052: Pseudahrensia
ASV3907: Methylacidiphilaceae
ASV0267: Thiotrichaceae
ASV1460: Candidatus Megaira
ASV0257: Micrococcaceae
ASV2990: LD29
ASV3532: Aeromonas eucrenophila
ASV2593: Pseudooceanicola
ASV4264: Burkholderiaceae
ASV2877: Clade I
ASV1622: Cyanobium PCC-6307
ASV0680: Amorphus
ASV3550: Mycobacterium
ASV4065: pltb-vmat-80
ASV2482: Thalassospira
ASV1545: Gammaproteobacteria
ASV4400: SAR116 clade
ASV2922: marine metagenome
ASV0840: Rhodobacteraceae
ASV0204: Rhodobacteraceae
ASV4013: Sphingomonadaceae
ASV0486: Clade I
ASV4187: Rubinisphaeraceae
ASV2477: Clade I
ASV1026: IS-44
ASV1950: Thiotrichaceae
ASV3967: Dechloromonas
ASV3485: Hyphomonas
ASV4402: Flavobacterium
ASV2562: Alphaproteobacteria
ASV3824: Lentimonas
ASV4195: Erythrobacter

Figure S4. ASVs with the highest mean feature importance values. The 50 ASVs with the highest mean feature importance values across all 20 metadata categories, showing random forest classification accuracy (top; blue scale), mean feature importance (left; yellow-green scale) and feature importance within each metadata category (main; black-purple-yellow scale), normalized within each ASV. The dendrogram shows Bray-Curtis distance between the metadata categories, using feature importance values for all ASVs. Plots for all metadata categories are shown in Supplementary Section 3 alongside plots for each metadata category across all taxonomic levels.

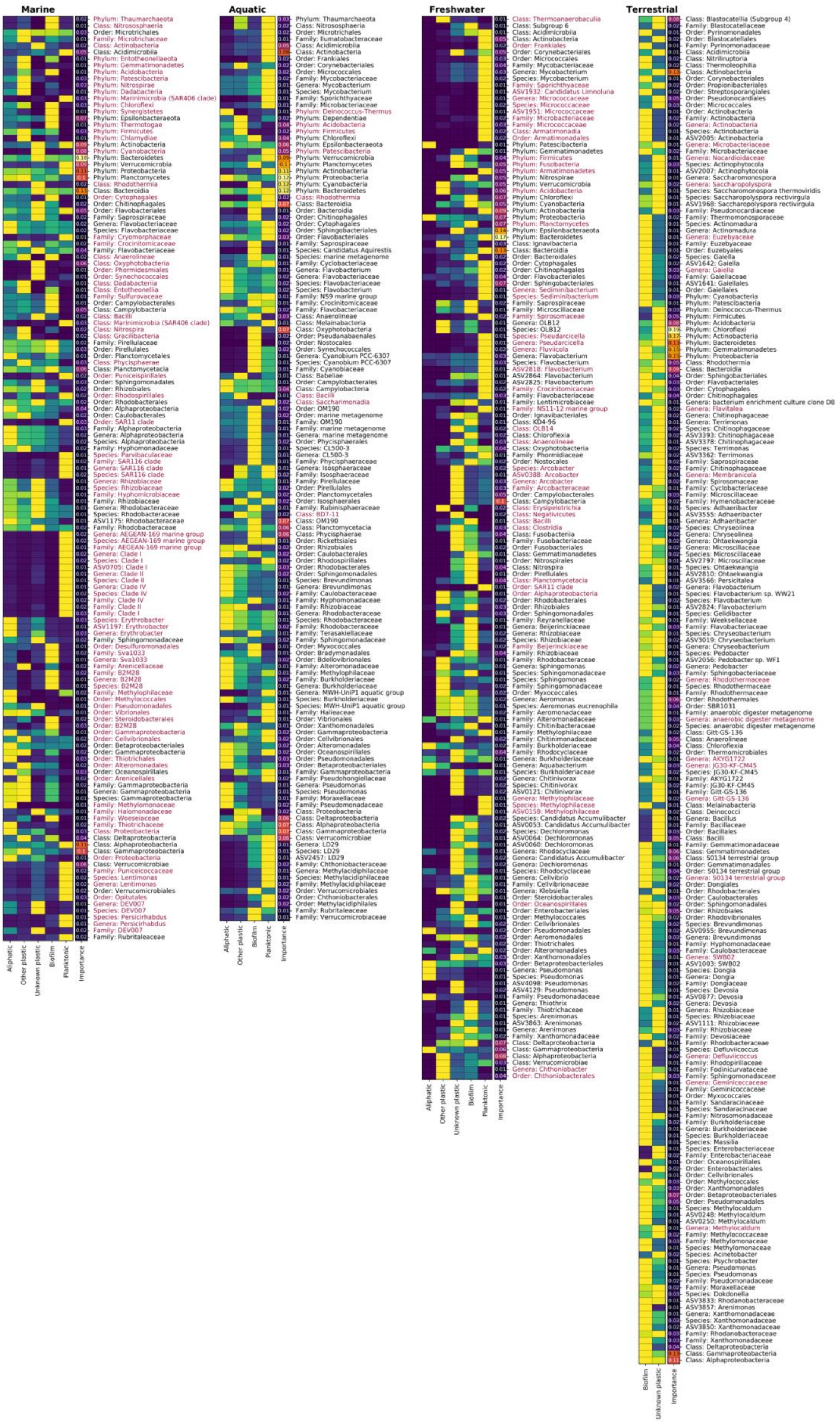
Marine
Aquatic
Freshwater
Terrestrial
Aliphatic
Other plastic
Unknown plastic
Biofilm
Planktonic
Importance

Figure S5. Important taxa for the random forest models constructed on Plastic type (general). Important taxa (feature importance values above 0.01 for all taxonomic levels; see Supplementary Section 3 for all taxa with feature importance values above 0.005) for the random forest classification models constructed on the Plastic type (general) metadata category for all marine, aquatic, freshwater and terrestrial samples, showing importance at all taxonomic levels (right, orange-pink-purple color scale) and abundance of the taxon in each substrate type (left, yellow-green-blue color scale for high-medium-low abundance). The names of taxa that were identified as significantly differentially abundant by ANCOM tests are shown in red, while others are shown in black.

Supplementary tables

Table S1. Summary of the studies returned by the literature searches, included studies and details of sample processing. All unique studies that were found by Web of Science Core Collection and Science Direct searches for the terms "Plastics plastisphere", "Plastics microbial community" and "Plastics microbial degradation" on 5th January 2020 and were manually assessed for inclusion in this study (A). All studies that met all of the inclusion criteria, details on whether the data was available, primers used and data processing details for all included studies as well as details on contact with the authors of the studies that could not be included (B). **This table is included as an excel file.**

Table S2. Metadata and accession numbers for all included samples. All meta-data collected for each included study, including sequence accession numbers and sample names within their respective studies. **This table is included as an excel file.**

Supplementary files and sections

Supplementary File 1. Instructions and code for reproducing all analyses in this manuscript or reproducing these analyses with additional studies.
This file can be found at: https://doi.org/10.6084/m9.figshare.12923855

Supplementary Section 1. Summary plots for each included study. Plots are shown that separately summarize each study that is included in the meta-analysis. Each page shows a separate study and contains nMDS plots (weighted and unweighted Unifrac distance) showing all samples as individual points (left; only those that had above 2000 reads remain here) as well the results of random forest models that were calculated for each study (right). These included all metadata categories that contained more than one grouping within that study. Classification accuracy for each of these is shown (top) and then the three metadata categories with the highest classification accuracy were chosen and the results of these are plotted alongside the 30 ASVs with the highest mean feature importance values and their relative abundance within each grouping (bottom). Where there were either no random forest models with a classification accuracy of above 0% or no metadata categories with more than one grouping in that study, the relative abundance of the 30 most abundant (mean) ASVs. Color bars are shown on the side for random forest classification accuracy, ASV importance and ASV relative abundance.
This section can be found at: https://doi.org/10.6084/m9.figshare.12233753

Supplementary Section 2. Full results for all four normalisation methods, including a discussion of the differences in significant taxa identified between these methods.
This section can be found at: https://doi.org/10.6084/m9.figshare.12915317

Supplementary Section 3. Random forest classification accuracy and important features. Random forest classification accuracy and important features across 20 metadata categories as well as four environments for general plastic type, each at all taxonomic levels.
This section can be found at: https://doi.org/10.6084/m9.figshare.12233759

Supplementary Section 4. Differential abundance between treatments and time points calculated by Metacoder. The figures shown in this section were all generated using the Metacoder package in R and show taxa that are significantly differentially abundant between two different groups (p<0.05; Wilcoxon rank sum tests with holm-bonferroni false discovery rate correction). The specific groupings that are compared in each plot are indicated on that page, alongside figure legends.
This section can be found at: https://doi.org/10.6084/m9.figshare.12233762

Supplementary Section 5. Differential abundance between treatments and time points calculated by ANCOM. Taxa that are differentially abundant at different time points as determined by ANCOM tests with holm-bonferroni false discovery rate correction. Each section shows comparisons within the same substrate at different time points and between substrates at the same time point. Only taxa that were significantly differentially abundant in at least one comparison are shown here and colors represent the treatment that that taxon was more abundant in.
This section can be found at: https://doi.org/10.6084/m9.figshare.12233765
